# Heart Failure: Epidemiology, Pathophysiology, and Management

**DOI:** 10.1002/mco2.70625

**Published:** 2026-03-14

**Authors:** Yujian Fan, Zhihua Yang, Qing Li, Meng Sun, Yumeng Pu, Ke Zhao, Yu Bao, Xianliang Wang, Jingyuan Mao, Zhiqiang Zhao

**Affiliations:** ^1^ First Teaching Hospital of Tianjin University of Traditional Chinese Medicine Tianjin China; ^2^ National Clinical Research Center for Chinese Medicine Acupuncture and Moxibustion Tianjin China; ^3^ School of Medicine The Chinese University of Hong Kong Shenzhen Guangdong China; ^4^ Guangzhou University of Chinese Medicine Guangzhou Guangdong China

**Keywords:** calcium homeostasis, calcium regulatory factors, calcium‐related pathways, heart failure, pathological mechanism

## Abstract

Heart failure (HF) is one of the leading causes of hospitalization and mortality worldwide. Despite continuous updates to modern clinical guidelines regarding HF classification and management, mortality and rehospitalization rates remain persistently high. Enhancing the prevention and treatment of HF therefore represents a major challenge both now and in the future. In this review, we synthesize HF epidemiology and systematically mapped the calcium‑handling differences under physiological and pathological conditions, as well as the patterns of calcium‑homeostasis dysregulation across the major HF subtypes (heart failure with reduced ejection fraction; heart failure with preserved ejection fraction). Moreover, we summarize alterations in key calcium‐regulating proteins (points) and calcium homeostasis‐related pathways (lines), and further integrate these nodes into a network model that links Ca^2^^+^ dynamics remodeling with inflammation, oxidative stress, and mitochondrial dysfunction. Building on this mechanistic network, we discuss both conventional HF management strategies and emerging therapeutic developments that target distinct mechanisms through points and lines. Finally, we outline the unmet needs and future directions across the current “diagnosis–treatment–monitoring” continuum, with the overarching goal of advancing precision diagnosis, individualized therapy, and the establishment of a comprehensive HF management framework.

## Graphical abstract

This article takes calcium homeostasis as the point of departure to provide an overview of heart failure from three perspectives—epidemiology, pathophysiological mechanisms, and management strategies—and summarizes the current unmet needs and future directions in the field.

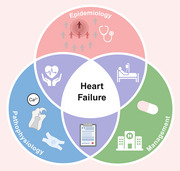

## 1 Introduction

According to the 2023 ESC guidelines [1], heart failure (HF) is categorized by left ventricular ejection fraction into three subtypes: heart failure with reduced ejection fraction (HFrEF), heart failure with mildly reduced ejection fraction (HFmrEF), and heart failure with preserved ejection fraction (HFpEF). The guidelines recommend sodium–glucose cotransporter 2 inhibitors (SGLT2i) for patients across the HF spectrum, highlighting their efficacy in reducing the risk of HF hospitalization and cardiovascular mortality. However, a “one‐size‐fits‐all” therapeutic strategy remains inadequate given the complex and heterogeneous nature of HF. Advancing more precise management approaches and identifying superior therapeutic agents require a deeper understanding of the fundamental pathophysiological mechanisms underlying HF.

The onset and progression of HF are influenced by multiple factors and are closely associated with mechanisms such as calcium homeostasis dysregulation, inflammation, oxidative stress, energy metabolism disturbances, cardiac fibrosis, and apoptosis [2, 3]. Among these, calcium homeostasis dysregulation is increasingly recognized as a pivotal determinant of both systolic and diastolic dysfunction in HF [4]. The 11th edition of major cardiology textbooks has also recognized calcium dysregulation as a key contributor to HF pathogenesis [5]. However, current research on calcium homeostasis dysregulation in HF predominantly focuses on the HFrEF [6]. Mechanistically, HFrEF is characterized by abrupt alterations in specific calcium‐handling proteins within the calcium cycling machinery—such as impaired sarcoplasmic reticulum (SR) Ca^2^^+^ reuptake or abnormal Ca^2^^+^ influx through L‐type calcium channels (LTCCs). These abnormalities lead to altered cytosolic Ca^2^^+^ amplitude, shortened or markedly delayed calcium transients (CaT), and ultimately impaired systolic and diastolic performance [7]. In contrast, studies dedicated to HFpEF—where diastolic dysfunction is the predominant feature—remain relatively scarce. Existing mechanistic investigations in HFpEF have largely focused on phenotype‐specific contributors such as obesity and hypertension [8]. With the recent redefinition of HF in clinical guidelines, comparative analyses between HFrEF and HFpEF have only recently gained attention [9, 10], yet most remain centered on hemodynamic differences rather than mechanistic validation, particularly regarding cytosolic, endoplasmic reticulum, and mitochondrial calcium dynamics.

Recent studies [11] have experimentally examined calcium‐regulating proteins across HF subtypes, suggesting that divergent patterns of calcium homeostasis dysregulation may underlie the differences between HFrEF and HFpEF. Additional research has highlighted mitochondrial calcium dysregulation as a key mechanism specifically implicated in HFpEF [12]. The “calcium homeostasis–systolic/diastolic function” may represent the core “mechanistic–functional” distinction among HF subtypes and serves as a key differentiator between HFrEF and HFpEF. These insights underscore the need for etiology‐specific therapeutic strategies tailored to the distinct pathophysiological profiles of HFrEF and HFpEF.

This study uses calcium homeostasis as a central framework to explore HF pathogenesis, moving from individual calcium‐regulating proteins (“points”) to integrated calcium homeostasis‐related signaling pathways (“lines”). It examines the differential roles of calcium homeostasis dysregulation in HFpEF and HFrEF, aiming to fill critical gaps in the mechanistic understanding of HF and to inform more targeted clinical strategies. Restoring calcium homeostasis—through modulation of calcium‐regulating proteins or calcium‐related signaling pathways—has demonstrated efficacy in improving both systolic and diastolic function [13, 14]. By elucidating the pivotal role of calcium homeostasis dysregulation in HF development, this study offers new perspectives and potential therapeutic avenues for HF prevention and treatment.

## 2 Epidemiology

HF is a rapidly escalating global public health concern, affecting more than 40 million individuals worldwide [15]. In several European countries, its prevalence among adults is approximately 1–2% [16]. While the prevalence of HFrEF has shown a gradual decline, HFpEF and HFmrEF continue to rise annually and now account for nearly half of all HF cases [17]. In Asian regions such as China, the incidence of HF is 275 per 100,000 person‑years (287 per 100,000 in men and 261 per 100,000 in women), with approximately 3 million new cases occurring annually [18]. The prognosis of patients with HF is poor, with mortality rates of 20 and 53% at 1 and 5 years after diagnosis, respectively [19]. Since 2012, HF‑related mortality has steadily increased, with a particularly sharp rise observed between 2020 and 2021. Age‑adjusted mortality rates in 2021 exceeded those reported in 1999 [20]. Compared with HFrEF, HFpEF has a higher burden of comorbidities and an increased rate of noncardiovascular mortality [21]. A registry‐based cohort study from heart failure centers in China in 2024 reported that cardiovascular death was the leading cause of mortality among patients with HF, accounting for 71.5% of all‑cause deaths. Moreover, patients with HFrEF and HFmrEF exhibited substantially higher mortality rates compared with those with HFpEF [22]. In the past, therapies effective for HFrEF have failed to demonstrate long‑term benefit in HFpEF [23]. These trends underscore the urgent need to elucidate the underlying pathophysiology of HF.

Cardiac diastole consists of two key phases: rapid ventricular filling (linked to isovolumic relaxation) and then late filling (linked to atrial contraction) [24]. The chief mechanism by which cardiac myocyte relaxation occurs is through ATP‐driven uptake of calcium by the SR via SR Ca^2+^ ATPase 2a (SERCA2a) and the dissociation of calcium from myofilaments [25]. Direct quantification of Ca^2^^+^ flux among major Ca^2^^+^‑handling proteins in nonfailing human cardiomyocytes has shown that SERCA2a accounts for 77% of the decline in intracellular Ca^2^^+^ concentration ([Ca^2^^+^]i), whereas the Na^+^/Ca^2^^+^ exchanger (NCX) contributes the remaining 23% [26]. In HFrEF, these proportions shift to 64% for SERCA2a and 36% for NCX, indicating a 57% increase in NCX's relative contribution [25]. Comparable high‑resolution analyses in HFpEF are still lacking. Accumulating data have indicated that protracted changes in workload promote not only remodelling of ventricular geometry, but also changes in T‐tubule structure within cardiomyocytes [11, 27, 28]. During HFrEF, loss and disorganization of T‐tubules impairs Ca^2+^ homeostasis, and thereby cellular contraction. However, similarly detailed investigations in HFpEF remain scarce.

Given the current landscape of HF, there is an urgent need to clarify its pathophysiology. This study adopts calcium homeostasis as the entry point to explore the “critical points,” “functional pathways,” and “mechanistic networks” that contribute to HF progression.

## 3 Pathophysiology

The heart is regulated by multiple, interacting mechanisms, including neurotransmitter signaling, energy metabolism, and mitochondrial function, which exhibit extensive crosstalk. For example, β‐adrenergic signaling, a key target in HF therapy, can exert anti‐inflammatory effects, improve endothelial function, and modulate ion channels [29]. Under physiological conditions, cardiac glucose and lipid metabolism are well balanced and mitochondria sustain continuous ATP production. However, when exposed to chronic pressure overload, hemodynamic stress, and altered myocardial perfusion, metabolic remodeling precedes structural remodeling in the heart [30]. Cardiac metabolic derangements are mainly driven by three mechanisms: (i) mitochondrial structural and functional abnormalities, (ii) altered substrate availability and utilization, and (iii) intracellular Ca^2^^+^ overload. Mitochondrial dysfunction is manifested by impaired respiration, organelle shrinkage or swelling, and loss of mitochondrial membrane potential, ultimately leading to reduced ATP synthesis. As mitochondrial structure and function deteriorate, substrate utilization is reprogrammed, resulting in an imbalance between fatty acid and glucose metabolism, decreased glucose oxidation, increased glycolysis, and consequent lactate accumulation. In addition, SERCA2a deficiency ultimately contributes to intracellular Na^+^ and Ca^2^^+^ overload [31].

Here, we use Ca^2^^+^ homeostasis as an entry point to examine the pathophysiological mechanisms across different HF phenotypes.

### 3.1 Calcium Regulating Proteins in Cardiac Physiology

**FIGURE 1:**
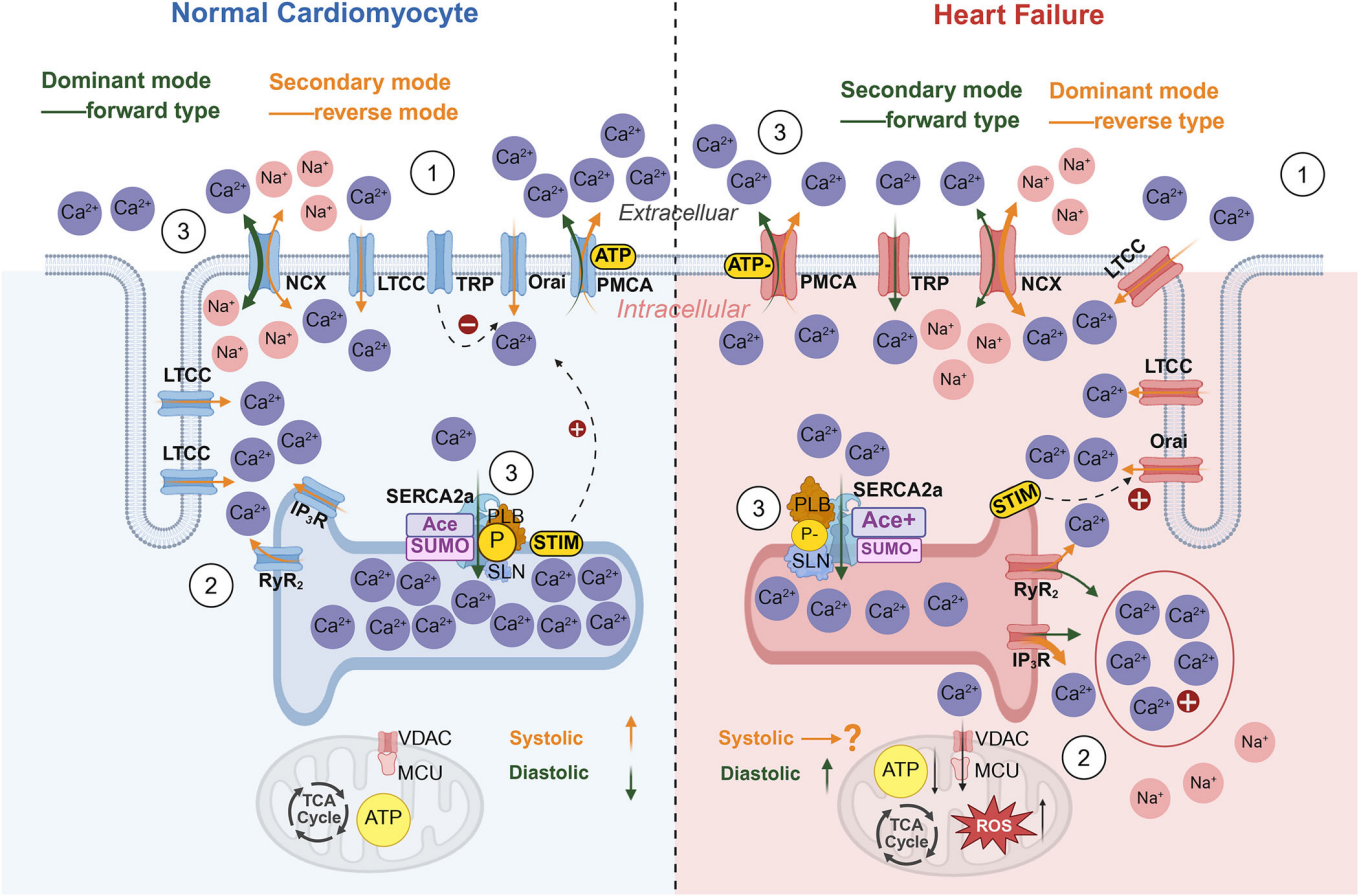
Mechanisms of calcium regulating proteins in physiological and pathological states (heart failure). All Ca^2+^ amounts in normal and HF were unified, where Ca^2+^ concentration in normal cardiomyocyte was as follows: SR > extracellular > cytoplasm, and Cart concentration in HF was as follows: cytoplasm > extracellular > SR. The flow of Ca^2+^ during systole/diastole is plotted, and the rising and falling trend of cytosolic Ca^2+^ during systole/diastole is indicated by arrows. Orange arrowheads indicate the direction of ion flux during systole, whereas green arrowheads denote the direction during diastole.

Calcium regulating proteins operate within a dynamic equilibrium and causal feedback loop. We describe their roles based on subcellular localization—extracellular space, cytosol, and SR—including components such as LTCCs and transverse tubule (T‐tube), NCX, plasma membrane calcium ATPase (PMCA), transient receptor potential (TRP), inositol‐trisphosphate receptor (IP_3_R), SERCA2a, phospholamban (PLN), sarcolipin (SLN), ryanodine receptor 2 (RyR_2_), and calcium release‐activated calcium channel (Orai), described across three phases: signal initiation, intracellular amplification, and calcium reuptake/recycling. Under normal physiological conditions, these calcium regulating proteins collectively maintain the systolic and diastolic function function of cardiomyocytes (Figure 1).

#### 3.1.1 Plasma Membrane: Extracellular‐to‐Cytosolic Ca^2^^+^ Flux

The working mode of NCX on the plasma membrane of cardiomyocytes during systolic period is mainly reversed, with Na^+^ being expelled from the cells and Ca^2+^ entering the cytoplasm (transport ratio is 3:1) [32]. LTCCs distribute on T‐tubes and normally open to promote Ca^2+^ entry into cytoplasm [33]. When intracellular Ca^2+^ increases, the affinity and transport rate of PMCA for Ca^2+^ increase, promoting Ca^2+^ excretion to cells—the so‐called “calcium influx promotes calcium excretion,” a negative feedback mechanism that maintains intracellular calcium homeostasis [34]. TRP is often in a closed state. When the intracellular Ca^2+^ concentration decreases, it triggers a physiological response of intracellular TRP opening [35]. When Ca^2+^ concentration increases to a certain extent, the negative feedback mechanism inhibits TRP opening and restores calcium homeostasis [36]. Moreover, some uncommon calcium regulating protein also play a non‐negligible role in cardiomyocytes. Hyperpolarization‐activated cyclic nucleotide‐gated (HCN) channel is activated when the cell membrane hyperpolarizes [37]. There are four types of HCN channels—HCN1, HCN2, HCN3, and HCN4—which are predominantly expressed in cardiac and neuronal cells [38] and play a key role in regulating the rhythmic activity of cellular networks [39]. The binding of cyclic adenosine monophosphate (cAMP) molecules and the CNBD region at the C‐terminal of the HCN channel enhances the inward current during diastolic If, causing depolarization of the sinus node membrane potential close to the threshold of Ca^2+^ channel activation, triggering action potentials, thereby maintaining the rhythmic release of excitement [40]. Cardiomyocyte diastole is a different story. The working pattern of NCX on the plasma membrane is positive: Ca^2+^ is expelled from the cells and Na^+^ enters the cytoplasm. PMCA decreased its affinity and transport rate for Ca^2+^ and decreased its efflux of Ca^2+^.

#### 3.1.2 Organelles: Cytosolic to Intracellular Organelles Ca^2^^+^ Transfer

*Sarcoplasmic Reticulum*. During the systolic phase of cardiomyocytes, the RyR_2_ and IP_3_R on SR are open, and Ca^2+^ is released into the cytoplasm in SR [41]. The myocardial T‐tube ensures that RyR_2_ and LTCCs on the plasma membrane are close to each other [42], and inositol 1,4,5‐triphosphate (IP3) initiates LTCCs influx thereby stimulating RyR_2_ to release more Ca^2+^ for optimal calcium‐induced calcium release (CICR) [43, 44]. In addition, IP_3_R activates the NCX reverse mode and increases the influx of Ca^2+^ into the cytoplasm, which is closely related to the “excitement” in the excitation–contraction coupling of the heart [45]. When IP_3_R and RyRs on SR interact, they simultaneously release Ca^2+^ into the cytoplasm. The stromal interaction molecule (STIM) on SR senses the depletion of Ca^2+^ in SR, thereby stimulating Orai to allow Ca^2+^ influx (the interaction between STIM and Orai occurs in a “point‐to‐point” manner) [46]. During diastole period, with the increase of Ca^2+^ in cytoplasm, calmodulin (CaM) was formed to activate TRPM4 and inhibit Ca^2+^ influx into Orai [47]. SERCA2a, when unaffected by PLN, SLN, or other modifications, remains in an open state under normal activity and expression, thereby facilitating the reuptake of cytoplasmic Ca^2+^ into the SR [48, 49].

*Mitochondria*. Related calcium regulating proteins on mitochondria also play a key role. For example, voltage‐dependent anion channel 1 is the main calcium ion transport channel on the outer membrane of mitochondria and participates in cellular metabolism by transporting ATP and other small metabolites, thereby regulating the TCA cycle and reactive oxygen generation [50]. The mitochondrial calcium uniporter (MCU) is located on the inner membrane of mitochondria [51]. It is influenced by mitochondrial calcium uptake 1 and mitochondrial calcium uptake 2 and can promote intracellular Ca^2+^ flow to mitochondria, thereby maintaining intracellular calcium homeostasis and oxygen free radical homeostasis [52].

### 3.2 Critical Points Linking Calcium Homeostasis Dysregulation to HF

**FIGURE 2:**
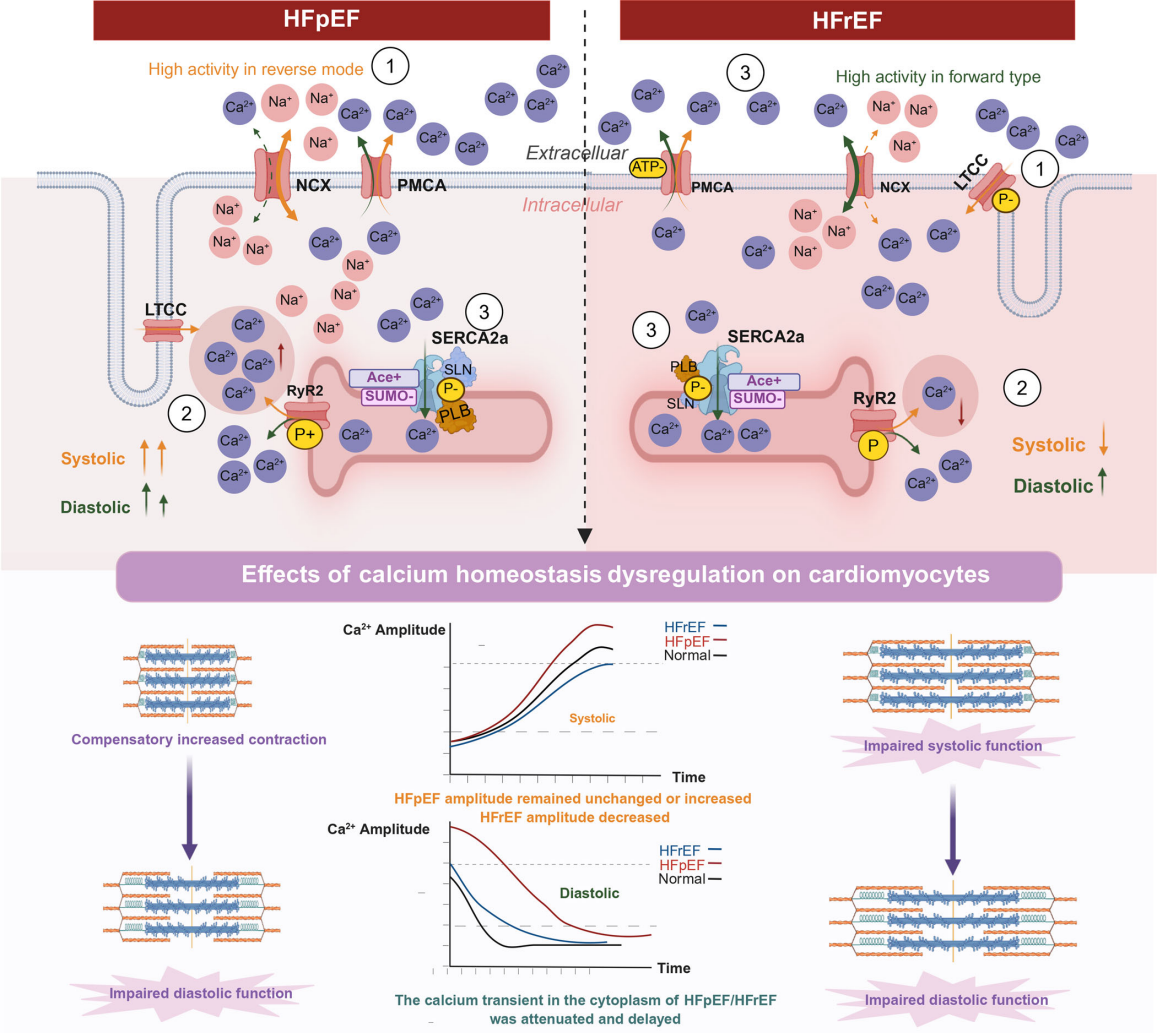
Pathological differences in calcium regulating proteins between HFrEF and HFpEF. At the molecular level, the forward and reverse modes of NCX are illustrated, together with the differential expression and posttranslational modifications of SERCA2a, RyR_2_, PMCA, and LTCC, as well as the distribution pattern of LTCC. At the functional level, CaT dynamics and sarcomeric states during systole and diastole are depicted. Orange arrowheads indicate the direction of ion flux during systole, whereas green arrowheads denote the direction during diastole.

There is currently no relevant research on HFmrEF calcium regulating proteins, and HFmrEF will not be included in the comparison range for the time being. (Figure 2 was constructed based on the pathological differences between HFrEF and HFpEF, and from a macroscopic perspective distinguishes the dynamic evolution of the normal state, HFrEF, and HFpEF.)

#### 3.2.1 Plasma Membrane: Extracellular‐to‐Cytosolic Ca^2^^+^ Flux

*PMCA* is an ATP‐driven Ca^2+^ pump capable of maintaining low Ca^2+^ concentrations at rest [53]. Four structurally similar PMCA subtypes have been identified in mammals (PMCA1–PMCA4), in which PMCA1 and PMCA4 are widely expressed without restriction [54]. PMCA has a CaM complex binding domain, which has an inhibitory effect on itself. When intracellular Ca^2+^ increases, more Ca–CaM complexes are formed, thus releasing the inhibition on PMCA and promoting Ca^2+^ efflux [55]. Several studies have shown that in both HFrEF and HFpEF, reduced expression or activity of PMCA impairs efficient Ca^2^^+^ extrusion, leading to delayed calcium clearance and contributing to cardiomyocyte systolic and diastolic dysfunction [56].

In HFrEF, the rate of Ca^2^^+^ extrusion via PMCA is reduced, whereas in HFpEF, the amount of Ca^2^^+^ expelled through PMCA is diminished [57]. In the future, specific experiments should be carried out to analyze the differences in PMCA expression and activity, so as to fill in the relevant mechanisms of calcium homeostasis in cardiomyocytes, which is helpful for further exploration in the field of HF.

*NCX* is a bidirectional ion transporter on the cardiomyocyte membrane [58]. Its main function is to use the potential energy of the sodium ion concentration gradient on both sides of the membrane established by sodium pump activity, and the direction of the current is consistent with the direction of sodium flow [59]. Sodium and calcium ions are exchanged on the cell membrane to maintain a low concentration of free calcium ions within the cell. The activity of NCX is regulated by many factors, including membrane potential, intracellular and extracellular calcium concentration, phosphorylation, and so on [60]. In HF, NCX expression and activity has always been a controversial issue. Despite differences in models and detection methods, the expression, activity, and predominant mode of NCX can yield completely opposite outcomes within the same disease context.

In HFrEF, Frisk suggested that there was no change in NCX activity [11], Rouhana showed a decrease in NCX activity [61], and Gupta experimental results showed an increase in NCX expression and phosphorylation [62]. Hu experiments showed that although NCX expression decreased, it was mainly in a positive high activity state, leading to sustained excretion of Ca^2+^, a decrease in intracellular Ca^2+^, and a contraction dysfunction [63].

In HFpEF, Frisk found increased NCX activity in ischemic and hypertensive types [11], and Rouhana found decreased NCX activity in deficient blood types [61]. Kamimura studies have shown that NCX is overexpressed and the negative mode activity increases (increased sodium ion concentration in myocardial cells reduces the forward type activity of NCX, enhances the negative type activity, and even directly converts to the reverse type) [64]. At present, NCX inhibitors are mostly applied to HFpEF [65]. In the future, the new trend of targeting drugs of different HF is to weaken the positive mode of NCX in HFrEF and the negative mode in HFpEF, so as to balance sodium and calcium homeostasis.

*LTCC and T‐Tube*: There are three voltage‐gated Ca^2+^ channels: Cav1, Cav2, and Cav3, of which Cav1 channels are also called LTCC. LTCC was first found in the heart and smooth muscle and includes four subtypes: Cav1.1, Cav1.2, Cav1.3, and Cav1.4 [66]. We know that these cardiovascular channels are almost entirely of the Cav1.2 subtype, and they are blocked by clinically used Ca^2+^ channel blockers such as nifedipine, amlodipine, verapamil, and diltiazem [67]. Mentioning LTCC has to mention the specific organelles on the myocardial cell membrane‐myocardial T‐tubes. Although T‐tubes are not proteins that regulate Ca^2+^, they are closely related to the coupling of cardiomyocyte excitation–contraction [68]. LTCC is rich in myocardial T‐tubes. Therefore, the density of T‐tubes is usually consistent with LTCC expression in cardiomyocytes and the intracellular calcium amplitude [69]. HF is typically characterized by T‐tube disorder, followed by impaired or redistribution of LTCC expression (mostly distributed on the ridge) [70, 71], eventually resulting in damage to CICR.

In HFrEF, reduced T‐tubule density and increased collagen deposition lead to downregulation of LTCC expression [11]. During systole, LTCC redistribution results in diminished Ca^2^^+^ influx from the extracellular space into the cytosol. This is accompanied by disruption of the spatial coupling between LTCCs and RyR_2_, forming isolated RyR_2_ domains [72]. Consequently, Ca^2^^+^ release from the SR is delayed, impairing CICR [73] and contributing to systolic dysfunction. However, other studies have shown that in HFrEF rats (induced by coronary artery ligation), LTCC protein expression remains unchanged, while LTCC phosphorylation is reduced. Since phosphorylation enhances LTCC open probability, open time, and conductance—thereby promoting Ca^2^^+^ influx—its reduction leads to decreased calcium entry, disrupts excitation–contraction coupling, and ultimately impairs systolic function [74].

In HFpEF, T‐tubule density is increased and the structures are dilated, while collagen deposition does not significantly infiltrate the T‐tubules [75]. Studies have shown that higher T‐tubule density and greater expansion are associated with impaired diastolic function. Among different HFpEF phenotypes, T‐tubule density follows a descending order: ischemic > hypertensive > diabetic [11]. In the hypertensive phenotype of HFpEF, LTCC expression is upregulated, leading to enhanced Ca^2^^+^ influx and excessive RyR_2_‐mediated Ca^2^^+^ release from the SR. This overactivation of CICR amplifies calcium amplitude, enhancing systolic function while concurrently impairing diastolic function [76].

*TRP* channels are a large family of nonselective cation channel proteins, with six isoforms families with similar sequences: TRPC, TRPM, TRPV, TRPA, TRPML, and TRPP (or PKD) [77]. In 1997, research found that capsaicin can activate the TRPV1 channel to allow Ca^2+^ to flow into the cell [78], and TRPV1 can also be inhibited from opening itself by intracellular Ca^2+^ binding with CaM.

In HFrEF, insufficient expression of TRPV1 may reduce myocardial contractility [79], whereas excessive TRPV1 expression can lead to mitochondrial calcium overload [80]. The increased expression and activity of TRPC channels activate the TLR‐mediated inflammatory signaling pathway, which facilitates Ca^2^^+^ leakage from the SR via RyR_2_ and IP_3_R, thereby impairing diastolic function [81, 82]. Low expression of TRPM channels contributes to cardiac hypertrophy and increased Ca^2^^+^ influx [47]. Despite their limited expression in cardiomyocytes, TRP channels have not been systematically investigated in the context of HFrEF versus HFpEF. Focused research on this channel may uncover novel pathophysiological mechanisms and address current gaps in HF characterization.

*Orai*. Store‐operated calcium entry (SOCE) is a ubiquitous mechanism for calcium signal generation and calcium homeostasis maintenance in animal cells [83]. SOCE is mediated by the STIM sensor on SR and the Orai on the plasma membrane [84]. In HF, excessive activation of SOCE or loss of SOCE results in immunodeficiency [85, 86].

In HFrEF, SOCE may be impaired. The reduced Ca^2^^+^ content in the SR fails to adequately activate STIM sensors, preventing the opening of Orai channels on the plasma membrane and thereby limiting Ca^2^^+^ influx into the cytosol [87].

In contrast, in HFpEF, SOCE may be excessively activated. The decreased SR Ca^2^^+^ content triggers STIM sensors on the SR to activate Orai channels on the plasma membrane, leading to enhanced Ca^2^^+^ influx into the cytosol. SOCE inhibitors, as immunosuppressants or anti‐inflammatory agents, can become new targets for drugs to treat HF [88].

#### 3.2.2 Organelles: Cytosolic to Intracellular Organelles Ca^2^^+^ Transfer

##### 3.2.2.1 Sarcoplasmic Reticulum

*SERCA2a* is a cardiac‐specific calcium pump located on the SR membrane. SERCA2a uses the energy from ATP hydrolysis to actively transport 75% of cytosolic Ca^2+^ into the SR during diastole, contributing to efficient cardiac relaxation and refilling of SR Ca^2+^ stores for subsequent contractions [89]. This process is inseparable from SERCA2a itself's activity, expression, posttranslational modification and regulation by transmembrane micropeptide [90]. Among them, ubiquitination, acetylation, and phosphorylation are the most important modification methods of SERCA2a. Considering the different modeling methods, most studies use the protein expression of SERCA2a/PLB and pPLB/PLB to reflect their functional roles [61]. A few studies used caffeine‐induced CaT to calculate the rate of Ca^2+^ reuptake and removal from cells to evaluate SERCA2a and NCX functions. Current studies have indicated that SERCA2a is a potential therapeutic target for both acute and chronic HF [91]. The activity of SERCA2a in HFrEF and HFpEF remains inconsistent, and no unified standard has been established to date.

Studies have shown that in HFrEF, both the expression and activity of SERCA2a are reduced, leading to impaired Ca^2^^+^ reuptake into the SR. Consequently, the rate of cytosolic Ca^2^^+^ clearance declines [11], CaT are attenuated, and myocardial contractility is diminished to a certain extent [90]. From a posttranslational modification perspective, decreased ubiquitination and phosphorylation, along with increased acetylation of SERCA2a, may contribute to its reduced activity [92]. Oxidation of SERCA2a at Cys498 leads to a decrease in its expression [93].

In HFpEF, the situation is more complex. In the ZSF‐1 rat model of HFpEF, which is based on diabetes and hypertension, SERCA2a expression is elevated, yet its activity is paradoxically decreased. Conversely, in HFpEF models induced by minor myocardial infarction, SERCA2a activity appears to be increased [94]. The direct role of SERCA2a dysfunction in HFpEF remains undetermined [25]. These experimental discrepancies likely arise from differences in disease modeling approaches, posttranslational modifications, and protein–protein interactions. Therefore, in addition to considering the subtype of HF, it is essential to account for the underlying pathogenic factors affecting SERCA2a when developing targeted pharmacological modulators.

*PLB and SLN*. exert inhibitory effects on SERCA2a, thereby reducing the capacity for Ca^2^^+^ reuptake into the SR and ultimately impairing cardiac systolic and diastolic function [95]. PLB is a calcium‐sensitive phosphoprotein; when phosphorylated (pPLB), its inhibitory effect on SERCA2a is attenuated, enhancing SERCA2a‐mediated Ca^2^^+^ uptake. In HFrEF, PLB expression is elevated while pPLB levels are decreased [76]. In HFpEF, PLB expression is also increased, yet the SERCA2a/PLB ratio is reduced [94]. In both HFrEF and HFpEF, the net effect is suppression of SERCA2a activity and impaired CaT dynamics. SLN is a low‐molecular‐weight protein homologous to PLB, sharing a similar transmembrane domain. Unlike PLB, SLN reduces Ca^2^^+^ accumulation within the SR without affecting ATP hydrolysis rate [96]. In HFrEF, SLN expression is downregulated, whereas SERCA2a expression is upregulated, leading to excessive SR Ca^2^^+^ loading and systolic dysfunction [97]. Whether SLN expression is elevated in HFpEF remains unclear due to a lack of definitive studies. Interestingly, SLN expression is significantly upregulated in diabetic HF models compared with nondiabetic counterparts [98].

*RyR_2_*. RyRs are Ca^2^^+^ release channels located on the SR of cardiomyocytes. There are three subtypes of RyR_1_, RyR_2_, and RyR_3_. Among them, impaired RyR_2_ opening during systole or aberrant activation during diastole can disrupt SR Ca^2^^+^ homeostasis, thereby contributing to HF or cardiac arrhythmias [99]. In HFrEF, RyR_2_ expression during systole is reduced or functionally impaired, resulting in insufficient Ca^2^^+^ release from the SR into the cytosol. This limits actomyosin cross‐bridge formation and severely compromises systolic function [100]. In HFpEF, RyR_2_ exhibits excessive opening during systole, leading to markedly greater Ca^2^^+^ efflux from the SR compared with HFrEF, which is closely associated with the compensatory enhancement of cardiomyocyte contractility in HFpEF [101]. However, other studies have reported that RyR_2_ expression levels are comparable between HFrEF and HFpEF, yet both conditions display hyperphosphorylation of RyR_2_, with HFpEF showing a higher degree of phosphorylation [11]. This posttranslational modification may underlie the aberrant diastolic opening of RyR_2_, disrupted CICR, and impaired diastolic function.

*IP_3_R* is a membrane glycoprotein complex located in the endoplasmic reticulum that requires a second messenger to be activated by IP_3_ [102]. Among the IP_3_R subtypes, there are two types related to cardiomyocytes: IP_3_R2 and IP_3_R3. Atrial and ventricular myocytes in most animals mainly express IP_3_R2 and a small number express IP_3_R3 [103]. Their distribution in the atrium is much larger than that in the ventricle. The structure and function of IP_3_R are regulated by multiple factors such as phosphorylation, protein interactions, or negative calcium ion feedback. In HFrEF, RyRs expression is downregulated, while IP_3_R may be upregulated as a compensatory response, providing an alternative pathway for intracellular Ca^2+^ [100]. In HFpEF, phospholipase C (PLC) in cardiomyocytes is activated during diastolic phase, and IP_3_ stimulates IP_3_R to release Ca^2+^ stored in SR [104], thereby increasing intracellular Ca^2+^ concentration, leading to an increase in related diastolic tension.

##### 3.2.2.2 Mitochondria

*MCU* is a highly selective Ca^2^^+^ channel located on the inner mitochondrial membrane, responsible for transporting cytosolic Ca^2^^+^ into the mitochondrial matrix [69]. In HFrEF, the expression level of MCU is compensated for increased, which may be to increase calcium uptake by mitochondria to maintain myocardial contractility and improve energy production [105].

**FIGURE 3:**
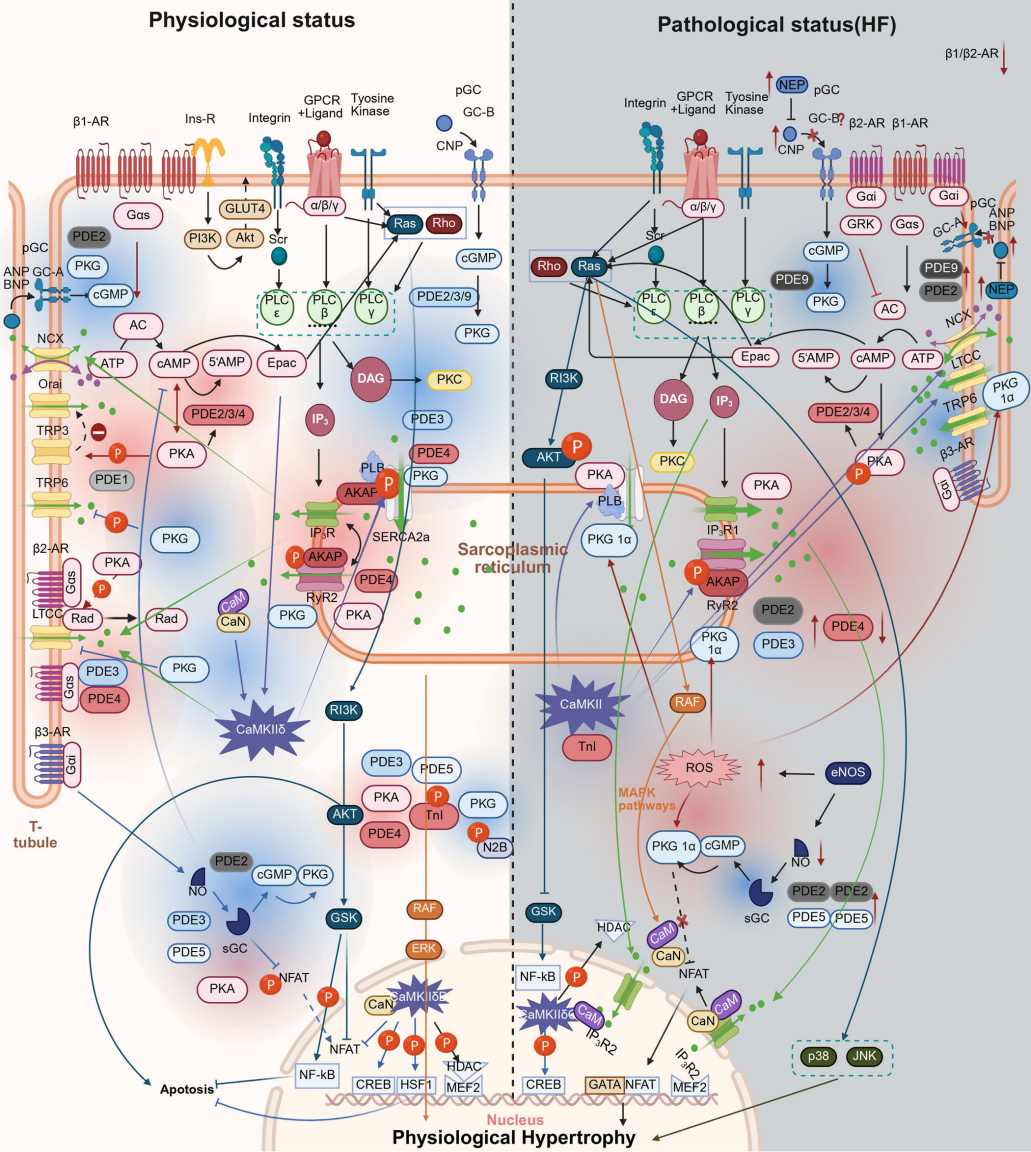
Mechanisms of calcium homeostasis‐related signaling pathways in physiological and pathological states (heart failure). The diagram illustrates the interactions of the cAMP, cGMP, CaMKII, RAS, and PI3K pathways with calcium regulating proteins and calcium ions. Blue highlights denote cGMP microdomains (GC‐A and GC‐B), whereas red highlights denote cAMP microdomains. Under physiological conditions, cGMP microdomains predominate over cAMP microdomains; in HF, this relationship is reversed. The balance between the two influences calcium regulating proteins. In addition, the figure depicts how the CaMKII, RAS, and PI3K pathways differentially contribute to physiological versus pathological cardiac hypertrophy.

Having identified calcium regulating proteins as critical points of calcium homeostasis disruption, we subsequently delineated the microdomain architecture, molecular homology classifications, and interrelationships among calcium homeostasis‐related signaling pathways (Figure 3).

### 3.3 Calcium Homeostasis‐Related Signaling Pathways in Cardiac Physiology

#### 3.3.1 cAMP Pathway

Neurohormones activate β‐adrenergic receptors on the plasma membrane to couple with heterologous triguanine nucleotide regulatory proteins (G proteins), causing βγ dimers to release active Gas and activating adenylcyclase (AC) to promote cAMP production [106]. The G protein‐coupled receptor kinases (GRK) family consists of seven members (GRK1–7), which can be classified into three subgroups based on gene structure and sequence homology: the visual or rhodopsin kinase subfamily (GRK1 and GRK7), the βAR kinase subfamily (GRK2 and GRK3), and the GRK4 subfamily (GRK4, GRK5, and GRK6) [107]. Among them, GRK2 and GRK5 are expressed in nearly all cardiac cell types [108, 109]. GRK2 phosphorylates activated βARs, which promotes the recruitment of β‑arrestins. This process uncouples the receptor from G proteins, leading to desensitization of βAR signaling [110, 111]. Similar to GRK2, overexpression of GRK5 leads to marked β‑adrenergic receptor desensitization [112]. Under physiological conditions, GRKs modulate the Ras signaling pathway, thereby influencing cardiac physiological hypertrophy [113].

This cAMP can be hydrolyzed by phosphodiesterases (PDEs). Among them, cAMP stimulated by β1‐AR is regulated by PDE2, PDE3, and PDE4, with a large microdomain. However, β2‐AR is regulated by PDE3 and PDE4, and the cAMP microdomain is restricted. cAMP and its downstream effector protein kinase A (PKA) are crucial biochemical messengers in regulating myocardial cell function. PKA can negatively feedback upstream AC and regulate stimulation of downstream PDEs. PKA is divided into multiple regions with the help of A‐kinase anchoring proteins [114]. Activation of the cAMP/PKA signaling pathway can directly phosphorylate several key proteins involved in excitation–contraction coupling, including the LTCC, PLB, RyR_2_, and troponin I (TnI) [115]. Short‐term activation of the cAMP pathway is beneficial to the heart. PKA phosphorylates guanosine 5′‐triphosphate (GTP)‐binding protein RAD, thereby enhancing Ca^2^^+^ influx—not through direct phosphorylation of LTCC. Activation of the cAMP pathway also facilitates systolic Ca^2^^+^ release by phosphorylating RyR_2_, accelerates Ca^2^^+^ reuptake into the SR via PLB phosphorylation and subsequent SERCA2a activation, and modulates myofilament sensitivity through phosphorylation of TnI. Additionally, short‐term activation of the cAMP pathway promotes HCN channel activity, contributing to the regulation of diastolic function.

#### 3.3.2 Cyclic Guanosine Monophosphate Pathway

Cyclic guanosine monophosphate (cGMP) is the second messenger that regulates various physiological processes such as cardiac contraction, vascular tone, and cardiac remodeling. GTP is catalyzed by guanosine cyclase (GC) to synthesize cGMP. Upstream of cGMP are two types of GCs: (1) natriuretic peptides (NPs) activate particulate (membrane bound) GC (NP–GC, also known as pGC). For example, purine cyclase A (GC‐A, also known as NPR1 or NPRA) is activated by ANP and BNP, and purine cyclase B (GC‐B, also known as NPR2 or NPRB) is activated by CNP; (2) nitric oxide (NO) activates soluble (intracellular) GC (NO‐GC, also known as sGC) [116]. pGC‐A is located in the T tubule and pGC‐B is distributed throughout the sarcolma. The GC‐A microdomain has little effect on contractility, while the GC‐B microdomain has a positive inotropic effect. The activity of GC‐A microdomain is regulated by PDE2 and PDE9, while GC‐B microdomain is also influenced by PDE3. The cGMP microdomain produced by sGC is generated through β_3_‐AR and is regulated by PDE5 and PDE3. PDE9 primarily hydrolyzes cGMP derived from the pGC pathway, whereas PDE5 mainly hydrolyzes cGMP generated via the sGC pathway. Under physiological conditions, the abundance of PDE2 in cardiomyocytes is relatively low. Binding of cGMP to the regulatory domain of PDE2 stimulates cAMP degradation, thereby attenuating excessive cAMP responses [117].

#### 3.3.3 Calcium/CaM‐Dependent Protein Kinase II Pathway

Calcium/CaM‐dependent protein kinase II (CaMKII) is a serine/threonine kinase comprising four distinct subtypes (α, β, γ, δ), with CaMKIIδ being the most prominent subtype in cardiomyocytes. CaMKIIδ is activated by Ca/CaM, reactive oxygen species (ROS), and exchange protein directly activated by cAMP. Activated CaMKIIδ produces a stimulating effect by phosphorylation of related calcium regulating proteins, such as increasing the inward current through LTCC and boosting the phosphorylation of PLB, which improves SR uptake of cytoplasmic Ca^2+^. In the nucleus, CaMKIIδB plays a protective role for the heart [118, 119]. CaMKIIδB mediates the phosphorylation of histone deacetylases (HDAC) and inhibits the transcription of the hypertrophy factor myocyte enhancer factor 2 (MEF‐2). Phosphorylation‐induced calcineurin (CaN), a regulator of the nuclear factors of activated T cells (NFAT) transcription factor involved in cardiac hypertrophy, also plays a role in transcription regulation [120]. CaMKIIδB increases phosphorylated heat shock factor 1 (HSF1), enhances intracellular heat shock protein 70 gene expression, and resists myocardial apoptosis [121]. Enhance MCU gene transcription by phosphorylated cAMP response element binding protein (CREB), facilitate the transfer of Ca^2+^ of SR to mitochondria, maintain intracellular calcium homeostasis, and prevent cardiac hypertrophy and diastolic dysfunction [122].

#### 3.3.4 Ras Pathway

This classical pathway of Ras is called “pathway drug cocktail” and used to be the preferred target of cancer [123]. Its downstream PLCε, JNK, and GSK can affect HF fibrosis, inflammation, oxidative stress, mitochondrial function, and metabolic disorders, and interact with calcium homeostasis. Small G protein of Ras family (guanosine triphosphate binding) consists of enzymes that hydrolyze GDP and interact with various tyrosine kinase receptors (EGF, PDGF, etc.) and GPCR [124]. ERK pathway downstream of Ras is associated with physiological hypertrophy of heart induced by long‐term exercise. Phosphatidylinositol 3‐kinase (PI3K) pathway can inhibit NFAT nuclear translocation and phosphorylate NF‐kB, which plays a dual role in promoting survival and antiapoptosis of heart.

#### 3.3.5 PI3K Pathway

PI3Ks protein family is involved in the regulation of multiple cellular functions such as cell survival, growth, metabolism and blood sugar homeostasis. Different PI3K isoforms have different effects.

PI3K γ stimulates cells to form PLC through G protein receptors [125]. Different isoforms of PLC can be activated by various upstream signals: PLCβ is activated by Gαq, PLCγ by receptor tyrosine kinases, and PLCε by Rho and Ras. PLC catalyzes the conversion of phosphatidylinositol 4,5‐bisphosphate (PIP2) into diacylglycerol (DAG) and IP_3_. DAG activates protein kinase C, while IP_3_ stimulates IP_3_R on SR. Notably, IP_3_R can colocalize with RyR_2_, allowing for mutual regulation and activation [126], which promotes calcium influx from the extracellular space (via LTCC and NCX), a process referred to as IP3‐induced calcium release.

PI3Kα phosphorylates PIP2 to produce PIP3, inhibiting sodium influx through the cytoplasmic Nav1.5 channel. Additionally, PI3Kα phosphorylates Akt, enhancing the phosphorylation of PLB, which increases SERCA2a activity and lowers intracellular Ca^2+^ levels. Inhibiting the expression and activity of IP_3_R reduces Ca^2+^ reaching mitochondria through MCU, reduces ROS, increases ATP, and further improves PMCA and SERCA2a activities. PI3Kβ inhibits phosphatase and tensin homolog, thereby reducing the dephosphorylation of PIP3 to PIP2.

### 3.4 Functional Lines Linking Calcium Homeostasis Dysregulation to HF

**FIGURE 4:**
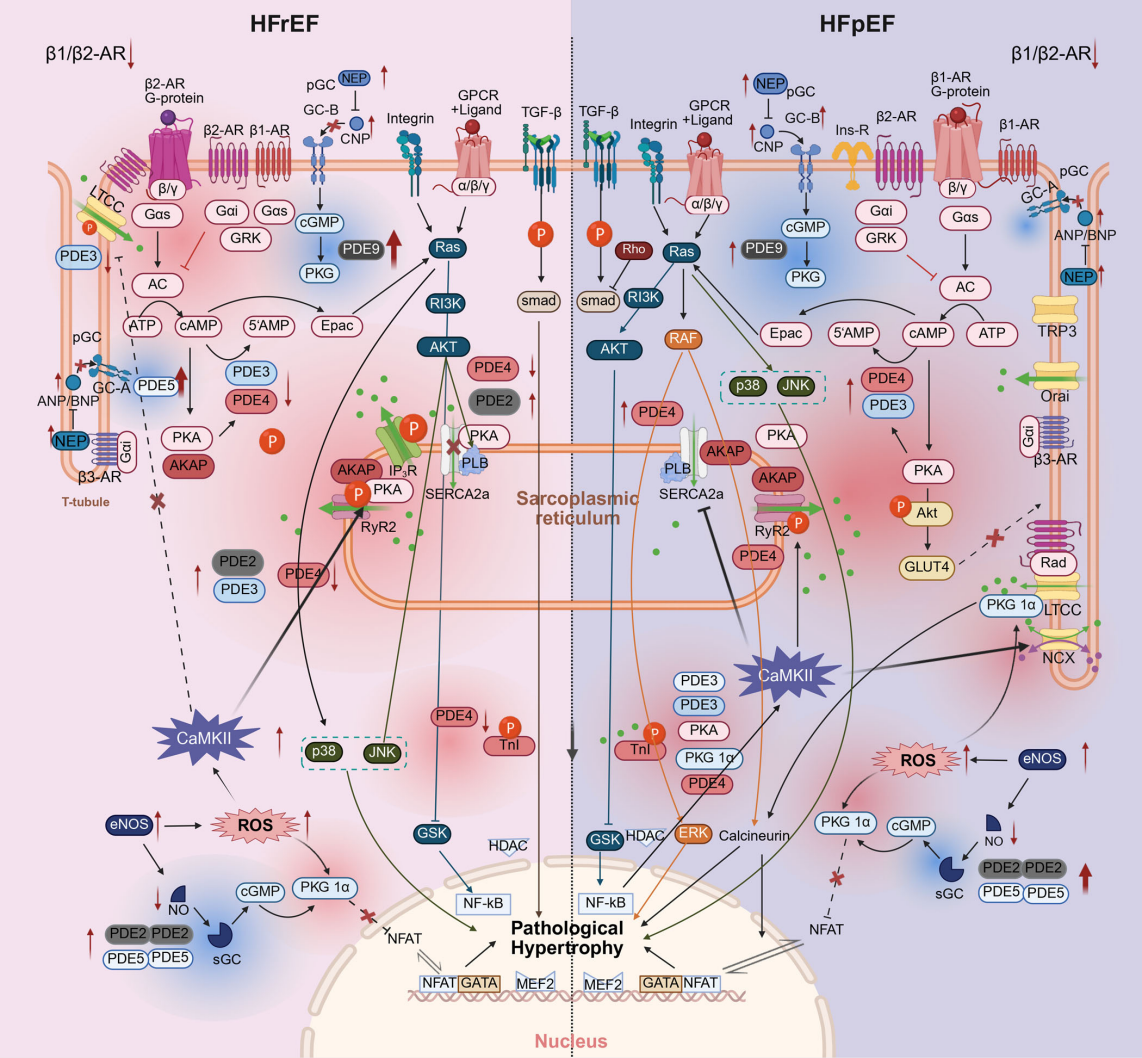
Pathological differences in calcium homeostasis‐related signaling pathways between HFrEF and HFpEF. The diagram depicts the differences in the cGMP pathway (GC‐A and GC‐B) between HFrEF and HFpEF, as well as the distinct interactions of the cAMP, CaMKII, Ras, and PI3K pathways with calcium regulating proteins.

We next aimed to identify the specific stages at which calcium dysregulation emerges during HF progression and to clarify the “functional lines” that drive this imbalance. This analysis also highlights the mechanistic distinctions between HFrEF and HFpEF (Figure 4).

#### 3.4.1 cAMP Pathway

Remodeling of cAMP microdomains is currently a major focus in HF research, while sustained activation of cAMP can trigger HF [127]. In HF, both β1‐AR and β2‐AR are downregulated, with a reduced β1‐AR/β2‐AR ratio and redistribution of each receptor subtype. The microdomains between cAMP and PDE are diminished, and Ca^2^^+^ flux is altered. HFrEF and HFpEF exhibit distinct patterns of cAMP microdomain organization.

In HFrEF, cAMP metabolite concentrations are elevated [128], with neurohormonal activation serving as the primary driver. β1‐AR sensitivity is reduced, whereas β2‐AR ionotropic signaling is enhanced. Gαi coupling and recruitment of GRKs are increased. Elevated GRK2 levels simultaneously induce hypertrophic gene expression, impair insulin signaling, and promote myocardial fibrosis [108]. GRK5 contributes to impaired cardiac function and immune cell recruitment [113]. Due to T‐tubule defects, β2‐AR and LTCCs are redistributed to the crests or tops of the sarcolemma. Within β2‐AR‐associated cAMP microdomains, PDE3 and PDE4 activity is reduced, leading to decreased LTCC phosphorylation. In β1‐AR‐associated cAMP microdomains, PDE2 and PDE3 activity is increased while PDE4 activity is reduced, resulting in RyR_2_ hyperphosphorylation and PLN hypophosphorylation [129]. Consequently, systolic intracellular Ca^2^^+^ is reduced, and myocardial contractility declines.

In HFpEF, cAMP metabolite concentrations are lower [128]. Comorbidities drive cAMP signaling through inflammation, increased ROS, and reduced NO bioavailability. In HFpEF, β2‐AR interacts with the insulin receptor, thereby promoting Gαi coupling and GRK recruitment. However, some studies have reported that GRK2 and GRK5 show no significant increase compared with normal controls [130]. Within β2‐AR‐associated cAMP microdomains, PDE3 and PDE4 expression is upregulated, leading to PLN hypophosphorylation and reduced SERCA2a activity [131]. This increases Ca^2^^+^ transient kinetics or elevates diastolic cytosolic Ca^2^^+^ concentration, ultimately causing diastolic dysfunction. PKA‐mediated Akt phosphorylation regulates glucose uptake; however, sustained Akt activation and reduced GLUT4 translocation contribute to insulin resistance.

#### 3.4.2 cGMP Pathway

In HF, although NEP and total NPs concentrations are increased, NPs are degraded at the cell surface by associated proteases, leading to a reduced ratio of mature (active) to immature (inactive) NPs and thereby diminished NP activation [132]. GC‐A activity is markedly reduced, whereas GC‐B activity remains unchanged or is increased [133]. Endothelial NO synthase generates pathological ROS, resulting in decreased NO and subsequent reduction of sGC activity. Expression of PDE2, PDE5, and PDE9 is upregulated, leading to enhanced hydrolysis of cGMP. PDE2 hydrolyzes both cGMP and cAMP, playing a critical role in crosstalk between the cGMP and cAMP pathways. At this stage, altered cGMP microdomain organization prevents cAMP degradation [134]. Downstream protein kinase G (PKG) is no longer activated but instead oxidized, while ROS directly reduce NO release from cardiovascular tissues.

In HFrEF, pGC‐associated cGMP microdomains are severely impaired. Expression of SERCA2a and pPLB is reduced, resulting in diminished SR Ca^2^^+^ uptake during diastole and elevated cytosolic Ca^2^^+^ [135]. This activates CaMKII, which alters LTCC distribution (restricted to crests), causing LTCC dysfunction and reduced systolic Ca^2^^+^ influx [136].

In HFpEF, pGC‐associated GC‐B microdomains are enhanced, whereas sGC‐associated cGMP microdomains are reduced [137]. Because sGC‐mediated NO activation is attenuated, inhibition of LTCC and TRPC6 is diminished, leading to increased systolic Ca^2^^+^ influx [138]. PLB phosphorylation is reduced, SR Ca^2^^+^ reuptake is impaired, and myofilament Ca^2^^+^ affinity is increased [139, 140], resulting in compensatory augmentation of contractile force in HFpEF [141]. However, Ca^2^^+^ activates CaN, which dephosphorylates NFAT, promoting its nuclear translocation and amplifying hypertrophic signaling [142]. This damages myofibrils and initiates a vicious cycle. Downstream PKG directly regulates diastolic function and is closely linked to HFpEF pathophysiology [143]. Nevertheless, isosorbide dinitrate, the only PKG modulator tested to date, worsens HFpEF‐related phenotypes, and other PKG‐targeted agents have not consistently improved outcomes in HF patients [144]. Future studies are warranted to further explore the cGMP signaling pathway.

#### 3.4.3 CaMKII Pathway

In HF, nuclear CaMKIIδB expression is reduced, whereas CaMKIIδC expression is increased [121]. CaMKIIδB‐mediated phosphorylation of CREB and HSF1 is attenuated, leading to decreased MCU transcription. In contrast, CaMKIIδC enhances HDAC phosphorylation and nuclear translocation, diminishes repression of MEF‐2 expression, and promotes nuclear NFAT–GATA binding, thereby inducing cardiac hypertrophy [120]. Autophosphorylation of CaMKIIδC also modulates the cAMP‐binding protein CREB; phosphorylated CREB upregulates IP_3_R1 expression, facilitating IP_3_R1‐mediated Ca^2^^+^ release.

In HFrEF, CaMKII is hyperactivated, markedly increasing RyR_2_ phosphorylation to a greater extent than PKA‐mediated phosphorylation. Phosphorylation of RyR_2_ promotes systolic SR Ca^2^^+^ release and diastolic SR Ca^2^^+^ leak, activating inward NCX currents [118] and thereby triggering depolarization.

In HFpEF, CaMKII expression is reduced, accompanied by decreased Thr17 phosphorylation of PLB, which weakens SR Ca^2^^+^ reuptake [145]. However, RyR_2_ phosphorylation is excessively increased, resulting in an imbalance between elevated NCX expression and reduced SERCA2a expression [146].

#### 3.4.4 Ras Pathway

In HF, chronic activation of Akt suppresses autophagy, while excessive activation of NF‐κB triggers inflammation and pro‐apoptotic signaling, both of which are key pathogenic factors [147]. Activation of the p38 and JNK pathways, as well as CaN downstream of Ras–RAF signaling, represent critical mediators of pathological hypertrophy. Clinically, RAS inhibitors are used more frequently in HFrEF than in HFpEF [148]; however, their application in basic experimental models is considerably more complex.

In HFrEF, the NF‐κB/p38/JNK pathway is activated [149]. JNK1 facilitates the interaction of Akt with PLB at Thr17, thereby inhibiting SERCA2a and preventing diastolic SR Ca^2^^+^ reuptake. Concurrently, the TGFβ/Smad pathway is activated, inducing inflammation and fibrosis, which further disrupt Ca^2^^+^ homeostasis.

In HFpEF, the Rho/ROCK pathway is upregulated, promoting actin–myosin contraction in cardiomyocytes. Rho/ROCK signaling suppresses Smad activity, thereby reducing TGFβ/Smad‐dependent signaling [150]. In addition, Ras/ERK signaling is upregulated, contributing to interstitial fibrosis. NF‐κB/p38/JNK signaling is also enhanced; increased NF‐κB activity stimulates CaMKII phosphorylation, which in turn promotes RyR_2_ phosphorylation and upregulation of NCX expression, leading to Ca^2^^+^ homeostatic imbalance.

#### 3.4.5 PI3K Pathway

In HF, increased PI3Kγ, together with IP_3_ generated by PLC and Ca^2^^+^ released from RyR_2_, jointly regulate IP_3_R2 located on the nuclear envelope, thereby triggering nuclear Ca^2^^+^ signaling. This activates HDAC and NFAT shuttling, stimulating transcription factors that induce gene hypertrophy and ultimately drive cardiac remodeling [151]. Nuclear Ca^2^^+^ signaling can also activate CaMKII, which in turn promotes LTCC opening. By contrast, the activities of PI3Kα and PI3Kβ are relatively diminished, impairing their dual roles in suppressing autophagy and promoting mitophagy.

In HFrEF, RyR_2_ expression is reduced, whereas IP_3_R expression is compensatorily increased, with phosphorylation of both RyR_2_ and IP_3_R elevated in parallel [152].

In HFpEF, no fundamental experimental studies have yet been conducted on this pathway. Beyond Ca^2^^+^ homeostasis, this pathway is implicated in mitochondrial metabolism, apoptosis, inflammation, and other processes closely linked to the heterogeneity of HFpEF. The key protein IP_3_R within this pathway has also emerged as a promising target in the exploration of HFpEF phenotypes associated with atrial fibrillation, making it a recent focus of research.

This section focuses on analyzing the signaling pathways most frequently studied in HF over the past 5 years under conditions of calcium homeostasis dysregulation. Crosstalk between the cAMP and cGMP pathways is evident: under normal conditions, activation of the cAMP pathway is lower than that of cGMP, whereas in HF, cAMP pathway activation exceeds that of cGMP. Dysregulation of these two pathways, accompanied by inflammation and oxidative stress, contributes to calcium homeostasis dysregulation. The CaMKII and Ras pathways represent interconnected signaling cascades linking extracellular, cytoplasmic, and nuclear compartments of cardiomyocytes. Ca^2^^+^ imbalance drives the CaMKII pathway to induce secondary mechanisms such as fibrosis and apoptosis, while Ras pathway abnormalities simultaneously provoke calcium homeostasis dysregulation, mitochondrial dysfunction, inflammation, and impaired autophagy. PI3K, as a downstream branch of the Ras pathway, primarily regulates calcium homeostasis between the cytoplasm and intracellular organelles. It remains difficult to delineate the temporal sequence among these mechanisms or pathways; instead, calcium homeostasis dysregulation can only be described in terms of its specific localization within cardiomyocytes or its significance within a given pathway. Identifying critical points within calcium homeostasis, linking them to functional lines, and constructing mechanistic networks to drive effective clinical therapies remains a major challenge.

### 3.5 Mechanistic Network of HF

**FIGURE 5:**
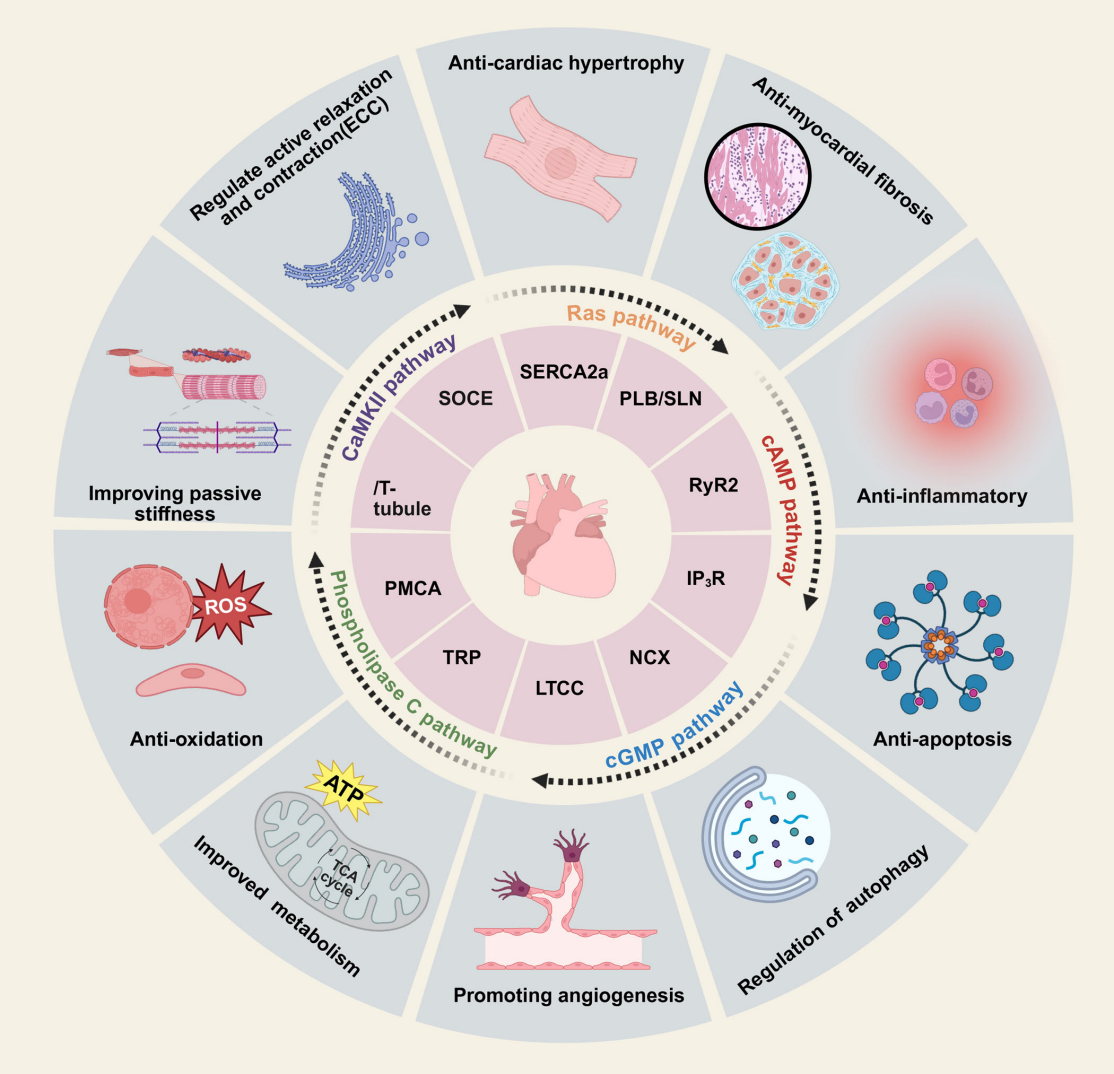
Pathophysiological mechanism network in heart failure. Starting from calcium homeostasis, calcium‐regulating proteins, or calcium‐related pathways interact with other mechanisms to jointly drive the onset and progression of heart failure.

As previously discussed, dysregulation of calcium homeostasis can arise from posttranslational modifications of calcium regulating proteins—for example, acetylation or phosphorylation of SERCA2a, which may alter its expression or activity, or shifts in the forward and reverse modes of NCX that influence the direction of Ca^2^^+^ flux. Changes in calcium‑related signaling pathways are often driven by the activation or relocalization of specific components within these pathways. Moreover, disturbances in calcium homeostasis may also result from crosstalk with other mechanisms, whereby parallel pathways modulate calcium‑associated signaling or inflammatory mediators modify calcium regulating proteins. In this context, we discuss the bidirectional causal interplay between calcium homeostasis and other regulatory mechanisms. Nevertheless, the fundamental nature of these processes remains under active investigation. Calcium homeostasis dysregulation is closely associated with multiple mechanisms—including cardiac fibrosis, mitochondrial dysfunction, inflammation, oxidative stress, cardiomyocyte hypertrophy, and stiffness (Figure 5)—that collectively drive the onset and progression of HF.

#### 3.5.1 Cyclic Reinforcement

Calcium homeostasis dysregulation interacts with fibrosis, mitochondrial dysfunction, oxidative stress, and inflammation in reinforcing cycles. Each mechanism amplifies the others, creating a self‑perpetuating pathological loop that accelerates HF progression.

Dysfunction of calcium regulating proteins in SR or abnormalities in the cAMP/cGMP signaling pathways lead to calcium homeostasis imbalance [153], thereby disturbing mitochondrial calcium regulation [154]. In HFrEF, mitochondrial calcium overload alters membrane potential and elevates ROS levels, triggering mitophagy and oxidative stress [155, 156], which subsequently contribute to cardiomyocyte hypertrophy [157]. In HFpEF, reduced mitochondrial calcium impairs ATP production and induces metabolic disturbances [158], resulting in passive stiffness. These reactions promote apoptosis [159], which in turn activates Ras or PI3K signaling pathways, driving inflammation [160]. Inflammation further induces fibrosis, thereby affecting CaMKII signaling and once again disrupting calcium homeostasis, ultimately impairing excitation–contraction coupling and establishing a vicious cycle [161].

#### 3.5.2 Dynamic and Bidirectional Interference

The relationship between calcium homeostasis dysregulation and other mechanisms is bidirectional and context dependent. Inflammation may arise as a consequence of calcium dysregulation yet also exacerbate it; oxidative stress both results from and worsens mitochondrial dysfunction. Such reciprocal coupling across pathways highlights the fluid and interconnected nature of the mechanistic web underlying HF.

In HFrEF, impaired calcium regulating proteins such as SERCA2a and RyR_2_ lead to cytosolic calcium overload. Excess calcium enters mitochondria, causing depolarization and ROS generation [162, 163]. The resulting oxidative stress further damages calcium cycling proteins [164], thereby reinforcing calcium homeostasis dysregulation in a bidirectional loop. In HFpEF, systemic inflammation and metabolic stress promote myocardial fibrosis and stiffness [165]. Increased stiffness impairs diastolic calcium reuptake, disturbing calcium homeostasis [166]. Calcium homeostasis dysregulation in turn activates inflammatory signaling pathways such as Ras pathway, which further drive fibrosis and stiffness [167, 168], forming a dynamic bidirectional interference.

Targeting the current pathophysiological mechanisms of HF provides opportunities for comprehensive patient management and the development of innovative therapeutic agents.

## 4 Management Strategies

At present, HF treatment still relies largely on conventional management strategies, including pharmacological and nonpharmacological interventions. The lack of individualized therapy has remained a persistent challenge in this field. In this section, we summarize guideline‐recommended management strategies for different HF phenotypes and, using calcium homeostasis as an illustrative example, review the current landscape of novel drug development, including both preclinical and clinical studies. To facilitate the future development of novel drugs directed at distinct mechanisms and molecular targets.

### 4.1 Current Standard Treatment: Holistic Comprehensive Management

Current management strategies for HF are individualized according to the type of HF and patient‐specific characteristics. Pharmacological therapy remains the cornerstone of HF management. For patients with HFrEF, guidelines recommend the use of diuretics, renin–angiotensin system inhibitors, β‑blockers, and mineralocorticoid receptor antagonists. For patients with HFimpEF, it is advised that guideline‐directed medical therapy (GDMT) be continued after improvement to prevent recurrence of HF and left ventricular dysfunction. For HFmrEF and HFpEF, diuretics and SGLT2i are recommended to alleviate symptoms and reduce the risk of hospitalization. Nonpharmacological interventions are integral throughout the course of HF management, including dietary regulation, exercise control, and device‐based therapies such as cardiac resynchronization therapy and implantable cardioverter‐defibrillators [169]. To prevent disease progression, dynamic monitoring of HF signs and biomarkers is required. Management of comorbidities such as hypertension, coronary artery disease, and atrial fibrillation is essential to reduce additional risk. Long‐term follow‐up and patient education are critical to improving adherence and optimizing outcomes.

### 4.2 Novel Horizons in Mechanism‑Oriented Treatment: Focusing on Calcium Homeostasis

Future development of pharmacological therapy is expected to follow a “critical point–functional line–mechanistic network” strategy to advance drug discovery for HF. Targeting calcium regulating proteins or calcium homeostasis‐related signaling pathways can alter ion flux direction and improve intracellular Na^+^–Ca^2^^+^ and acid–base balance in cardiomyocytes, thereby modulating excitation–contraction coupling and restoring both diastolic and systolic cardiac function.

To date, pharmacological agents targeting calcium regulating proteins or calcium homeostasis‐related signaling pathways in both HFrEF and HFpEF have been shown to effectively modulate pathological states [170]. Potential therapeutic targets for HFpEF include SERCA2a, NCX, PLB, and the Ras pathway [171]. Preclinical and clinical studies of calcium regulating proteins and calcium homeostasis‐related signaling pathways in HFrEF and HFpEF are summarized in Table 1, with classification according to molecular mechanisms.

**TABLE 1:** Novel therapeutic targets in HF via calcium homeostasis.

Medicine name	Mechanism	Research type	Animal	Proteins/pathways	HF type	References
Probenecid	Calcium homeostasis dysregulation, apoptosis, excitation–contraction coupling	Cell experiment	Primary cardiacmyocyte (C57)	TRP	HFrEF	[172]
Danicamtiv	Calcium homeostasis dysregulation, mitochondrial function, passive stiffness	Cell experiment	Skinned muscle fibers and myofibrils	Titin	HFrEF	[173]
Dantrolene	Calcium homeostasis dysregulation, metabolic disorder, oxidative stress	Cell experiment	Primary cardiacmyocyte (C57)	RyR_2_	HFpEF	[174]
Schaftoside	Calcium homeostasis dysregulation, autophagy, myocardial hypertrophy, inflammation	Cell experiment	AC16 cardiacmyocyte	CaMKII pathway	HFpEF	[175]
Methyltransferase‐like protein 13 (Mettl13)	Calcium homeostasis dysregulation, oxidative stress, fibrosis	Animal experiment	C57BL/6 mice	SERCA2a	HFrEF	[176]
PST3093 derivatives	Calcium homeostasis dysregulation, mitochondrial function	Animal experiment	SD rats	SERCA2a	HFrEF	[177]
Sinapic acid	Calcium homeostasis dysregulation, inflammation, oxidative stress, myocardial fibrosis	Animal experiment	Wistar rats	Ras pathways	HFrEF	[178]
Rhynchophylline	Calcium homeostasis dysregulation, mitochondrial function, apoptosis, oxidative stress	Animal experiment	C57BL/6 mice	RyR_2_, PLB, pPLB	HFrEF	[179]
Dantrolene sodium	Calcium homeostasis dysregulation, oxidative stress	Animal experiment	Guinea pig	RyR_2_	HFrEF	[180]
Luteolin	Calcium homeostasis dysregulation, fibrosis, excitation–contraction coupling	Animal experiment	SD rats	SERCA2a, NCX, PLB, PI3k pathway	HFrEF	[181]
Stachydrine hydrochloride	Calcium homeostasis dysregulation, myocardial hypertrophy, oxidative stress	Animal experiment	C57BL/6J mice	CaMKII pathway, LTCC, RyR_2_	HFrEF	[182]
CCG258208	Calcium homeostasis dysregulation, mitochondrial function, metabolic imbalance	Animal experiment	C57Bl/6 mice	cAMP pathway	HFrEF	[183]
Empagliflozin	Calcium homeostasis dysregulation, metabolic disorder	Animal experiment	C57Bl/6 mice	NHE	HFrEF	[184]
Periplocin	Calcium homeostasis dysregulation, inflammation, oxidative stress, myocardial fibrosis	Animal experiment	SD rats	Ras pathways	HFpEF	[185]
Nicotinamide	Calcium homeostasis dysregulation, metabolic imbalance, passive stiffness	Animal experiment	Leptin receptor‐deficient ZSF1 rats	SERCA2a, Titin	HFpEF	[186]
Berberine	Calcium homeostasis dysregulation, mitochondrial function, autophagy	Animal experiment	C57BL/6J male mice	PLB, SERCA2a	HFpEF	[187]
Triiodothyronine	Calcium homeostasis dysregulation, metabolic imbalance, fibrosis	Animal experiment	ZSF1 obese rats	RyR_2_	HFpEF	[188]
Acyl ghrelin	Calcium homeostasis dysregulation, metabolic imbalance, passive stiffness	Clinic trial	Human	Titin, cAMP pathway	HFrEF	[189]
Adenosine A1‐receptor agonist neladenoson bialanate	Calcium homeostasis dysregulation, metabolic imbalance	Clinic trial	Human	SERCA2a, Titin	HFrEF	[190]
AAV1/SERCA2a	Calcium homeostasis dysregulation	Clinic trial	Human	SERCA2a	HFrEF	[191]
β3‐Adrenoceptor Agonist	Calcium homeostasis dysregulation	Clinic trial	Human	NCX	HFrEF	[192]
Probenecid	Calcium homeostasis dysregulation, excitation–contraction coupling	Clinic trial	Human	TRPV	HFrEF	[193]
Tranilast	Calcium homeostasis dysregulation, inflammation	Clinic trial	Human	TRPV	HFrEF	[194]
CCBs	Calcium homeostasis dysregulation	Clinic trial	Human	LTCC	HFpEF	[195]

#### 4.2.1 Preclinical Experiments

Studies conducted in primary mouse cardiomyocytes and cardiomyocyte lines have identified several agents with potential therapeutic effects. For example, Probenecid and Danicamtiv target calcium regulating proteins, ameliorating the reduction of cytosolic calcium during systole in HFrEF, thereby restoring calcium homeostasis and modulating mitochondrial function [172, 173]. Schaftoside acts on calcium homeostasis‐related signaling pathways to improve delayed diastolic CaT in HFpEF, while also repairing mechanisms involving autophagy and inflammation [175]. Animal experiments have demonstrated that compounds such as Mettl13 and Luteolin can target calcium regulating proteins including SERCA2a, NCX, and PLB, enhancing SR Ca^2^^+^ reuptake during diastole, attenuating CaT in HFrEF, and regulating fibrosis as well as excitation–contraction coupling [176, 181]. The novel GRK2 inhibitor CCG258208, derived from paroxetine, improves left ventricular contractile function and limits adverse remodeling in HFrEF [183]. Berberine and Triiodothyronine, by targeting RyR_2_ and PLB, improve SR Ca^2^^+^ release during systole in HFpEF, augment CaT, and ameliorate fibrosis and mitochondrial dysfunction [187, 188]. Certain drugs regulate the activity or expression of calcium regulatory proteins by influencing their posttranslational modifications [93].

#### 4.2.2 Clinic Trials

In clinical trials, the calcium channel blockers (CCBs) familiar to us act as calcium homeostasis modulators targeting LTCC. Evidence indicates that CCBs can restore disrupted calcium homeostasis, thereby improving clinical symptoms and prognosis in patients with HFpEF [195]. Acyl ghrelin and the adenosine A1‑receptor agonist neladenoson bialanate, on the other hand, target calcium regulating proteins such as SERCA2a and Titin, as well as calcium homeostasis‐related signaling pathways including cAMP, thereby influencing metabolism and cardiomyocyte stiffness in HFrEF [189, 190]. As a RyR_2_ inhibitor, M201‐A has been shown to enhance natriuresis and improve renal function in humans, suggesting its potential as a therapeutic target for HF [196]. Pharmacological agents that inhibit RyR_2_ phosphorylation may also hold potential for improving HFpEF. At present, clinical studies focusing on specific calcium‑related targets or pathways remain at an early stage.

Western pharmacotherapy is supported by a relatively complete modern theoretical framework and robust clinical evidence, whereas natural medicines exert therapeutic effects through multicomponent, multipathway, and multitarget mechanisms. Combination therapy has opened innovative avenues in pharmacokinetics. In the near future, advances in modern scientific technologies, building upon prior research, may enable precise targeting of calcium regulating proteins or calcium homeostasis‐related signaling pathways to restore calcium homeostasis, facilitate drug development, modulate the pathological networks of HF, and generate additional clinical and experimental evidence.

## 5 Unmet Needs and Future Directions

Beyond establishing personalized management strategies for HF, several critical yet often overlooked challenges persist. Current diagnostic pathways are relatively rigid, and the supporting research and technical infrastructure is still insufficient, underscoring an urgent need for further optimization and strengthening. Prospectively, increased financial investment, strengthened experimental capacity, improved interdepartmental coordination, and deeper international collaboration will be essential for establishing a more resilient HF diagnostic–therapeutic system and a comprehensive safety framework.

### 5.1 Current Problems and Challenges in HF

Reducing mortality, decreasing rehospitalization rates, and improving quality of life remain urgent clinical priorities. Achieving these goals requires not only comprehensive mechanistic exploration but also methodological innovation. Traditional single‑omics approaches face challenges of reproducibility and standardization, with substantial variability observed across laboratories, analytical platforms, and study populations [197]. As a result, validating omics‑derived biomarkers in independent patient cohorts and translating these findings into clinically actionable diagnostic tools remain difficult, limiting their clinical utility. The inherent complexity of biological systems further complicates efforts to accurately model cardiac physiology [198]. Current heart‑on‑chip platforms fall short in replicating the heart's highly intricate multicellular architecture and spatial organization [199]. In parallel, dissemination and implementation of HF management guidelines remain suboptimal, with marked disparities in diagnostic accuracy and therapeutic practices across countries, regions, and healthcare institutions [200].

### 5.2 Future Directions

Advances in emerging fields such as epigenetics, stem cell transplantation, and metabolomics may represent important regulatory factors influencing the onset of HF [201]. Systems biology approaches offer a holistic framework for elucidating how calcium‑regulating proteins shape the complex biological networks underlying HF and how these networks drive disease evolution. High‑precision 3D printing technologies, compared with traditional two‑dimensional cultures, demonstrate superior physiological relevance and enhanced biological functionality [202]. Pluripotent stem cell‑based biomimetic heart systems hold great potential for high‑throughput screening in drug development and toxicity assessment [203]. Clinically, large‑scale, multicenter trials are needed to generate high‑quality evidence. From a management perspective, it is not only essential to establish rigorous evidence‑based guidelines but also to strengthen oversight at the primary care level and to promote the dissemination of HF knowledge across schools, hospitals, and the general population [204].

## 6 Conclusion and Prospect

An expanding body of research underscores the pivotal role of calcium homeostasis in cardiovascular physiology and disease. Disruptions in calcium balance interact with multiple pathological processes, including mitochondrial dysfunction, dysregulated apoptosis and autophagy, and impaired energy metabolism [205, 206]. Notably, accumulating evidence indicates that such disturbances directly contribute to muscle dysfunction in cardiomyocytes [170]. Targeting specific calcium regulating proteins or calcium homeostasis‐related pathways may represent potential therapeutic strategies for HF.

Calcium antagonists that target LTCCs have been clinically validated as effective [207, 208]. Modulating SERCA2a expression at the genetic, protein, and posttranslational levels also represents a compelling approach to enhance SERCA2a‑based therapies for HF management [209]. Moreover, structural damage to T‑tubules further highlights calcium homeostasis as an independent pathogenic factor in both HFrEF and HFpEF [11]. Elucidating the mechanistic distinctions between these HF subtypes and developing therapeutic interventions aimed at correcting T‑tubule abnormalities may therefore offer new opportunities for subtype‑specific treatment.

Despite these advances, clinical studies directly targeting calcium homeostasis‐related signaling pathways for HF prevention and therapy remain lacking. Current research on calcium dysregulation in HFrEF versus HFpEF is fragmented, and methodological approaches are still evolving. Existing investigations have yet to bridge the gap between the “critical points” of individual calcium‑regulating proteins and the broader “functional lines” of calcium‑related signaling pathways. Furthermore, the inability to clearly define the initiation nodes of calcium homeostasis dysregulation in HF has hindered the transition from “functional lines” to “mechanistic networks” in deeper exploration.

These challenges underscore the need for interdisciplinary research strategies. Traditional ex vivo experiments and animal models alone are insufficient to fully capture the dynamic alterations in calcium‑regulating proteins across HF subtypes. Integrating multiomics technologies—including transcriptomics, proteomics, and metabolomics—with advanced imaging modalities may enable more direct visualization of the spatiotemporal features of calcium homeostasis dysregulation [210, 211]. In parallel, artificial intelligence and systems biology offer powerful tools for identifying key regulatory nodes and pathways within complex datasets, thereby accelerating target discovery and drug development [212]. Strengthening clinical translational research is equally essential, as the application of calcium‑related targets and therapeutics to patient stratification, prognostic assessment, and personalized treatment remains unresolved [213]. Future efforts should prioritize multicenter clinical trials to explore the predictive value of calcium homeostasis‐related biomarkers across different HF phenotypes and to assess the safety and efficacy of calcium‐targeted therapies in real‐world patient populations. Only through bidirectional integration of basic and clinical research can calcium homeostasis studies be effectively translated into HF prevention and treatment.

A comprehensive focus on the dynamic equilibrium of calcium homeostasis—together with its interplay with key pathological processes such as inflammation and metabolic dysfunction [214]—may facilitate the establishment of a more systematic pathological network model of HF. Clarifying the specific characteristics of different HF phenotypes, prioritizing calcium‐targeted drugs, and conducting classification‐based research with detailed therapeutic strategies hold great potential for supplementing the pathological mechanisms of HF, addressing the current clinical challenges of HFpEF, and accelerating novel drug development. Ultimately, these efforts will contribute to improved patient outcomes, enhanced cardiovascular risk prevention, and more effective management of heart disease.

## Author Contributions

YF and ZY performed the literature search, selected relevant articles, interpreted data, and wrote the report. QL revised the manuscript. MS, YP, KZ, and YB performed the literature search. ZZ, XW, and JM designed and supervised this work. YF and ZY contributed equally to this work and shared the first authorship. All authors have read and approved the final submission.

## Funding Information

This work was supported by Tianjin Municipal Health Commission's second batch of high‐level talent selection and training project in the health industry “Jinmen Medical Talents” (No: TJSJMYXYC‐D2‐052), the “Mechanism Research of Yangyin Shuxin Formula Inhibits Myocardial Calcium Overload via the PI3K/IP_3_R Pathway to Improve Diastolic Function in Heart Failure with Preserved Ejection Fraction” of Scientific research projects in critical field of traditional Chinese medicine in Tianjin (No: 2024003).

## Ethics Statement

The authors have nothing to report.

## Conflicts of Interest

The authors declare no conflicts of interest.

## Data Availability

All data generated and/or analyzed during the current study are included in this published article.

## References

[1] T. A. McDonagh , M. Metra , M. Adamo , et al., “2023 Focused Update of the 2021 ESC Guidelines for the Diagnosis and Treatment of Acute and Chronic Heart Failure,” The European Heart Journal 44, no. 37 (2023): 3627–3639. 37622666 10.1093/eurheartj/ehad195

[2] J. Wen , L. Zhang , H. Liu , et al., “Salsolinol Attenuates Doxorubicin‐Induced Chronic Heart Failure in Rats and Improves Mitochondrial Function in H9c2 Cardiomyocytes,” Frontiers in pharmacology 10 (2019): 1135. 31680945 10.3389/fphar.2019.01135 PMC6797600

[3] Z. Liu , Y. Gan , Z. Shen , et al., “Role of Copper Homeostasis and Cuproptosis in Heart Failure Pathogenesis: Implications for Therapeutic Strategies,” Frontiers in pharmacology 15 (2024): 1527901. 39850564 10.3389/fphar.2024.1527901 PMC11754225

[4] Q. Guo , J. Wang , R. Sun , et al., “Comprehensive Construction of a Circular RNA‐Associated Competing Endogenous RNA Network Identified Novel Circular RNAs in Hypertrophic Cardiomyopathy by Integrated Analysis,” Frontiers in Genetics 11 (2020): 764. 32849787 10.3389/fgene.2020.00764 PMC7399352

[5] D. P. Zipes , P. Libby , and R. O. Bonow , Braunwald's Heart Disease: A Textbook of Cardiovascular Medicine (Elsevier/Saunders, 2019).

[6] D. Lei , Y. Liu , Y. Liu , et al., “The Gut Microbiota Metabolite Trimethylamine N‐oxide Promotes Cardiac Hypertrophy by Activating the Autophagic Degradation of SERCA2a,” Communications Biology 8, no. 1 (2025): 596. 40210720 10.1038/s42003-025-08016-9 PMC11986001

[7] L. G. Dias , C. H. O. Reis , L. Dos Santos , et al., “Strength Training Improves Heart Function, Collagen and Strength in Rats With Heart Failure,” The Journal of Physiological Sciences 74, no. 1 (2024): 10. 38365576 10.1186/s12576-024-00899-3 PMC10873996

[8] C. Withaar , L. M. G. Meems , G. Markousis‐Mavrogenis , et al., “The Effects of Liraglutide and Dapagliflozin on Cardiac Function and Structure in a Multi‐hit Mouse Model of Heart Failure With Preserved Ejection Fraction,” Cardiovascular Research 117, no. 9 (2021): 2108–2124. 32871009 10.1093/cvr/cvaa256 PMC8318109

[9] D. Roman‐Pepine , A. M. Serban , R. D. Capras , et al., “A Comprehensive Review: Unraveling the Role of Inflammation in the Etiology of Heart Failure,” Heart Failure Reviews 30, no. 5 (2025): 931–954. 40360833 10.1007/s10741-025-10519-w PMC12297024

[10] K. Yeh , M. Ansar , H. Jamal , et al., “A Retrospective Comparative Study of Sex‐Based Risk Variations in Heart Failure with Preserved Ejection Fraction (HFpEF) versus Heart Failure with Reduced Ejection Fraction (HFrEF),” Journal of Primary Care and Community Health 16 (2025): 21501319251367840. 10.1177/21501319251367840 PMC12374113 40842410

[11] M. Frisk , C. Le , X. Shen , et al., “Etiology‐Dependent Impairment of Diastolic Cardiomyocyte Calcium Homeostasis in Heart Failure with Preserved Ejection Fraction,” Journal of the American College of Cardiology 77, no. 4 (2021): 405–419. 33509397 10.1016/j.jacc.2020.11.044 PMC7840890

[12] M. Abudureyimu , X. Luo , L. Jiang , et al., “FBXL4 protects Against HFpEF Through Drp1‐Mediated Regulation of Mitochondrial Dynamics and the Downstream SERCA2a,” Redox Biology 70 (2024): 103081. 38359748 10.1016/j.redox.2024.103081 PMC10878117

[13] F. Tang , H. Han , S. Fu , et al., “Nonpharmacological Approaches to Managing Heart Failure with Preserved Ejection Fraction,” Circ Heart Fail 17, no. 8 (2024): e011269. 38887946 10.1161/CIRCHEARTFAILURE.123.011269 PMC11332382

[14] Y. Liu , J. Luo , L. Peng , et al., “Flavonoids: Potential Therapeutic Agents for Cardiovascular Disease,” Heliyon 10, no. 12 (2024): e32563. 38975137 10.1016/j.heliyon.2024.e32563 PMC11225753

[15] B. Wen , M. Liu , X. Qin , et al., “Identifying Immune Cell Infiltration and Diagnostic Biomarkers in Heart Failure and Osteoarthritis by Bioinformatics Analysis,” Medicine 102, no. 26 (2023): e34166. 37390254 10.1097/MD.0000000000034166 PMC10313258

[16] Y. Zhang , C. Gao , S. J. Greene , et al., “Clinical Performance and Quality Measures for Heart Failure Management in China: The China‐Heart Failure Registry Study,” ESC Heart Failure 10, no. 1 (2023): 342–352. 36224725 10.1002/ehf2.14184 PMC9871659

[17] S. Ambrosini , E. Gorica , S. A. Mohammed , et al., “Epigenetic Remodeling in Heart Failure With Preserved Ejection Fraction,” Current Opinion in Cardiology 37, no. 3 (2022): 219–226. 35275888 10.1097/HCO.0000000000000961 PMC9415220

[18] H. Wang , K. Chai , M. Du , et al., “Prevalence and Incidence of Heart Failure among Urban Patients in China: A National Population‐Based Analysis,” Circ Heart Fail 14, no. 10 (2021): e008406. 34455858 10.1161/CIRCHEARTFAILURE.121.008406

[19] T. A. McDonagh , M. Metra , M. Adamo , et al., “2021 ESC Guidelines for the Diagnosis and Treatment of Acute and Chronic Heart Failure,” European Heart Journal 42, no. 36 (2021): 3599–3726. 34447992 10.1093/eurheartj/ehab368

[20] G. C. Fonarow , F. S. Ahmad , T. Ahmad , et al., “HF STATS 2025: Heart Failure Epidemiology and Outcomes Statistics an Updated 2025 Report From the Heart Failure Society of America,” *Journal of Cardiac Failure*. 10.1016/j.cardfail.2025.07.007 40987671

[21] F. R. Heinzel and S. J. Shah , “The Future of Heart Failure With Preserved Ejection Fraction,” Herz 47, no. 4 (2022): 308–323. 35767073 10.1007/s00059-022-05124-8 PMC9244058

[22] H. Wang , Y. Li , K. Chai , et al., “Mortality in Patients Admitted to Hospital With Heart Failure in China: A Nationwide Cardiovascular Association Database‐Heart Failure Centre Registry Cohort Study,” The Lancet Global Health 12, no. 4 (2024): e611–e622. 38485428 10.1016/S2214-109X(23)00605-8

[23] I. Lakhani , K. S. K. Leung , G. Tse , et al., “Novel Mechanisms in Heart Failure with Preserved, Midrange, and Reduced Ejection Fraction,” Front Physiol 10 (2019): 874. 31333505 10.3389/fphys.2019.00874 PMC6625157

[24] K. Komamura , “Similarities and Differences Between the Pathogenesis and Pathophysiology of Diastolic and Systolic Heart Failure,” Cardiology Research and Practice 2013 (2013): 1–6. 10.1155/2013/824135 PMC3891535 24459600

[25] A. K. Shooshtarian , K. O'Gallagher , A. M. Shah , et al., “SERCA2a dysfunction in the Pathophysiology of Heart Failure With Preserved Ejection Fraction: A Direct Role Is yet to be Established,” Heart Failure Reviews 30, no. 3 (2025): 545–564. 39843817 10.1007/s10741-025-10487-1 PMC11991975

[26] V. Piacentino 3rd , C. R. Weber , X. Chen , et al., “Cellular Basis of Abnormal Calcium Transients of Failing human Ventricular Myocytes,” Circulation Research 92, no. 6 (2003): 651–658. 12600875 10.1161/01.RES.0000062469.83985.9B

[27] M. Ibrahim , P. Kukadia , U. Siedlecka , et al., “Cardiomyocyte Ca2+ Handling and Structure Is Regulated by Degree and Duration of Mechanical Load Variation,” Journal of Cellular and Molecular Medicine 16, no. 12 (2012): 2910–2918. 22862818 10.1111/j.1582-4934.2012.01611.x PMC4393719

[28] S. A. Watson , J. Duff , I. Bardi , et al., “Biomimetic Electromechanical Stimulation to Maintain Adult Myocardial Slices in Vitro,” Nature Communications 10, no. 1 (2019): 2168. 10.1038/s41467-019-10175-3 PMC6520377 31092830

[29] M. Kempiński , P. Jańczak , A. Porębska , et al., “Therapeutic Potential of the β3‐Adrenergic Receptor and Its Ligands in Cardiovascular Diseases,” The International Journal of Molecular Sciences 26, no. 24 (2025): 11844. 41465273 10.3390/ijms262411844 PMC12732782

[30] V. S. Hahn , C. Petucci , M. S. Kim , et al., “Myocardial Metabolomics of Human Heart Failure with Preserved Ejection Fraction,” Circulation 147, no. 15 (2023): 1147–1161. 36856044 10.1161/CIRCULATIONAHA.122.061846 PMC11059242

[31] Y. Fan , Z. Yang , L. Wang , et al., “Traditional Chinese Medicine for Heart Failure With Preserved Ejection Fraction: Clinical Evidence and Potential Mechanisms,” Frontiers in pharmacology 14 (2023): 1154167. 37234711 10.3389/fphar.2023.1154167 PMC10206212

[32] C. J. M. van Opbergen , L. den Braven , M. Delmar , et al., “Mitochondrial Dysfunction as Substrate for Arrhythmogenic Cardiomyopathy: A Search for New Disease Mechanisms,” Front Physiol 10 (2019): 1496. 31920701 10.3389/fphys.2019.01496 PMC6914828

[33] L. Liu , K. Zhou , X. Liu , et al., “The Interplay Between Cardiac Dyads and Mitochondria Regulated the Calcium Handling in Cardiomyocytes,” Front Physiol 13 (2022): 1013817. 36531185 10.3389/fphys.2022.1013817 PMC9755166

[34] Z. Cheng , Y. Liu , M. Ma , et al., “Lansoprazole‐induced Osteoporosis via the IP3R‐ and SOCE‐mediated Calcium Signaling Pathways,” Molecular Medicine 28, no. 1 (2022): 21. 35183103 10.1186/s10020-022-00448-x PMC8858482

[35] E. J. Behringer and M. A. Hakim , “Functional Interaction Among K(Ca) and TRP Channels for Cardiovascular Physiology: Modern Perspectives on Aging and Chronic Disease,” The International Journal of Molecular Sciences 20, no. 6 (2019): 1380. 30893836 10.3390/ijms20061380 PMC6471369

[36] N. Manolios , J. Papaemmanouil , and D. J. Adams , “The Role of Ion Channels in T Cell Function and Disease,” Frontiers in Immunology 14 (2023): 1238171. 37705981 10.3389/fimmu.2023.1238171 PMC10497217

[37] W. Parichatikanond , R. Duangrat , H. Kurose , et al., “Regulation of β‐Adrenergic Receptors in the Heart: A Review on Emerging Therapeutic Strategies for Heart Failure,” Cells 13, no. 20 (2024): 1674. 39451192 10.3390/cells13201674 PMC11506672

[38] W. Cai , S. S. Liu , B. M. Li , et al., “Presynaptic HCN Channels Constrain GABAergic Synaptic Transmission in Pyramidal Cells of the Medial Prefrontal Cortex,” Biology Open 11, no. 3 (2022): bio058840. 34709375 10.1242/bio.058840 PMC8966777

[39] D. Liu , A. T. Song , X. Qi , et al., “Cohesin‐protein Shugoshin‐1 Controls Cardiac Automaticity via HCN4 Pacemaker Channel,” Nature Communications 12, no. 1 (2021): 2551. 10.1038/s41467-021-22737-5 PMC8100125 33953173

[40] I. Shemarova , “The Dysfunction of Ca2+ Channels in Hereditary and Chronic Human Heart Diseases and Experimental Animal Models,” International Journal of Molecular Sciences 24, no. 21 (2023): 15682. 37958665 10.3390/ijms242115682 PMC10650855

[41] M. Dubois , D. Boulghobra , G. Rochebloine , et al., “Hyperglycemia Triggers RyR2‐dependent Alterations of Mitochondrial Calcium Homeostasis in Response to Cardiac Ischemia‐reperfusion: Key Role of DRP1 Activation,” Redox Biology 70 (2024): 103044. 38266577 10.1016/j.redox.2024.103044 PMC10835010

[42] F. Kermani , M. Mosqueira , K. Peters , et al., “Membrane Remodelling Triggers Maturation of Excitation‐contraction Coupling in 3D‐shaped human‐induced Pluripotent Stem Cell‐derived Cardiomyocytes,” Basic Research in Cardiology 118, no. 1 (2023): 13. 36988697 10.1007/s00395-023-00984-5 PMC10060306

[43] S. Ather , J. L. Respress , N. Li , et al., “Alterations in Ryanodine Receptors and Related Proteins in Heart Failure,” Biochimica et Biophysica Acta (BBA)—Molecular Basis of Disease 1832, no. 12 (2013): 2425–2431. 23770282 10.1016/j.bbadis.2013.06.008 PMC3800473

[44] H. L. Roderick and B. C. Knollmann , “Inositol 1,4,5‐trisphosphate Receptors: “Exciting” Players in Cardiac Excitation‐contraction Coupling?,” Circulation 128, no. 12 (2013): 1273–1275. 23983251 10.1161/CIRCULATIONAHA.113.005157 PMC3885819

[45] J. Kockskämper , A. V. Zima , H. L. Roderick , et al., “Emerging Roles of Inositol 1,4,5‐trisphosphate Signaling in Cardiac Myocytes,” Journal of Molecular and Cellular Cardiology 45, no. 2 (2008): 128–147. 18603259 10.1016/j.yjmcc.2008.05.014 PMC2654363

[46] H. Wen , J. K. Gwathmey , and L. H. Xie , “Role of Transient Receptor Potential Canonical Channels in Heart Physiology and Pathophysiology,” Frontiers in Cardiovascular Medicine 7 (2020): 24. 32158769 10.3389/fcvm.2020.00024 PMC7052113

[47] M. Kecskés , G. Jacobs , S. Kerselaers , et al., “The Ca2+‐activated Cation Channel TRPM4 Is a Negative Regulator of Angiotensin II‐induced Cardiac Hypertrophy,” Basic Research in Cardiology 110, no. 4 (2015): 43. 26043922 10.1007/s00395-015-0501-x PMC4456993

[48] T. Zhu , W. Zhang , Q. Yang , et al., “Effect of Angiotensin Receptor‐neprilysin Inhibitor on Atrial Electrical Instability in Atrial Fibrillation,” Frontiers in Cardiovascular Medicine 9 (2022): 1048077. 36568557 10.3389/fcvm.2022.1048077 PMC9772445

[49] Y. Zhai , J. Chen , R. Kan , et al., “B‐Type Natriuretic Peptide Inhibits the Expression and Function of SERCA2a in Heart Failure,” The International Journal of Cardiology 65, no. 2 (2024): 292–299. 10.1536/ihj.23-144 38556337

[50] H. Hu , L. Guo , J. Overholser , et al., “Mitochondrial VDAC1: A Potential Therapeutic Target of Inflammation‐Related Diseases and Clinical Opportunities,” Cells 11, no. 19 (2022): 3174. 36231136 10.3390/cells11193174 PMC9562648

[51] C. Chen , X. Dong , W. Zhang , et al., “Dialogue Between Mitochondria and Endoplasmic Reticulum‐potential Therapeutic Targets for Age‐related Cardiovascular Diseases,” Frontiers in pharmacology 15 (2024): 1389202. 38939842 10.3389/fphar.2024.1389202 PMC11208709

[52] M. Fan , J. Zhang , C.‐W. Tsai , et al., “Structure and Mechanism of the Mitochondrial Ca2+ Uniporter Holocomplex,” Nature 582, no. 7810 (2020): 129–133. 32494073 10.1038/s41586-020-2309-6 PMC7544431

[53] T. Boczek , M. Sobolczyk , J. Mackiewicz , et al., “Crosstalk Among Calcium ATPases: PMCA, SERCA and SPCA in Mental Diseases,” International Journal of Molecular Sciences 22, no. 6 (2021): 2785. 33801794 10.3390/ijms22062785 PMC8000800

[54] M. Takvam , C. M. Wood , H. Kryvi , et al., “Ion Transporters and Osmoregulation in the Kidney of Teleost Fishes as a Function of Salinity,” Front Physiol 12 (2021): 664588. 33967835 10.3389/fphys.2021.664588 PMC8098666

[55] B. Allegrini , M. Mignotet , R. Rapetti‐Mauss , et al., “A New Regulation Mechanism for KCNN4, the Ca(2+)‐dependent K(+) Channel, by Molecular Interactions With the Ca(2+)Pump PMCA4b,” The Journal of Biological Chemistry 301, no. 2 (2025): 108114. 39716493 10.1016/j.jbc.2024.108114 PMC11787511

[56] J. Borlak and T. Thum , “Hallmarks of Ion Channel Gene Expression in End‐stage Heart Failure,” The FASEB Journal 17, no. 12 (2003): 1592–1608. 12958166 10.1096/fj.02-0889com

[57] E. J. Cartwright , D. Oceandy , C. Austin , et al., “Ca2+ signalling in Cardiovascular Disease: The Role of the Plasma Membrane Calcium Pumps,” Science China Life Sciences 54, no. 8 (2011): 691–698. 21786192 10.1007/s11427-011-4199-1

[58] Y. Shemer , L. N. Mekies , R. Ben Jehuda , et al., “Investigating LMNA‐Related Dilated Cardiomyopathy Using Human Induced Pluripotent Stem Cell‐Derived Cardiomyocytes,” The International Journal of Molecular Sciences 22, no. 15 (2021): 7874. 34360639 10.3390/ijms22157874 PMC8346174

[59] T. Bombardini , “Myocardial Contractility in the Echo Lab: Molecular, Cellular and Pathophysiological Basis,” Cardiovascular ultrasound 3 (2005): 27. 16150150 10.1186/1476-7120-3-27 PMC1242240

[60] K. M. Haizlip and P. M. Janssen , “In Vitro Studies of Early Cardiac Remodeling: Impact on Contraction and Calcium Handling,” Front Biosci (Schol Ed) 3, no. 3 (2011): 1047. 21622254 10.2741/209 PMC3713459

[61] S. Rouhana , C. Farah , J. Roy , et al., “Early Calcium Handling Imbalance in Pressure Overload‐induced Heart Failure With Nearly Normal Left Ventricular Ejection Fraction,” Biochimica et Biophysica Acta (BBA)—Molecular Basis of Disease 1865, no. 1 (2019): 230–242. 30463691 10.1016/j.bbadis.2018.08.005

[62] R. C. Gupta , S. Mishra , M. Wang , et al., “Cardiac Contractility Modulation Electrical Signals Normalize Activity, Expression, and Phosphorylation of the Na+‐Ca2+ Exchanger in Heart Failure,” Journal of Cardiac Failure 15, no. 1 (2009): 48–56. 19181294 10.1016/j.cardfail.2008.08.011

[63] S. T. Hu , G. S. Liu , Y. F. Shen , et al., “Defective Ca(2+) Handling Proteins Regulation During Heart Failure,” Physiological Research 60, no. 1 (2011): 27–37. 20945956 10.33549/physiolres.931948

[64] D. Kamimura , T. Ohtani , Y. Sakata , et al., “Ca2+ entry Mode of Na+/Ca2+ Exchanger as a New Therapeutic Target for Heart Failure With Preserved Ejection Fraction,” European Heart Journal 33, no. 11 (2012): 1408–1416. 21490055 10.1093/eurheartj/ehr106

[65] M. Trum , J. Riechel , E. Schollmeier , et al., “Empagliflozin Inhibits Increased Na Influx in Atrial Cardiomyocytes of Patients With HFpEF,” Cardiovascular Research 120, no. 9 (2024): 999–1010. 38728438 10.1093/cvr/cvae095 PMC11288740

[66] E. G. Neureiter , M. Q. Erickson‐Oberg , A. Nigam , et al., “Inhibition of NMDA Receptors and Other Ion Channel Types by Membrane‐associated Drugs,” Frontiers in pharmacology 16 (2025): 1561956. 40371334 10.3389/fphar.2025.1561956 PMC12075551

[67] J. Striessnig , H. J. Bolz , and A. Koschak , “Channelopathies in Cav1.1, Cav1.3, and Cav1.4 Voltage‐gated L‐type Ca2+ Channels,” Pflügers Archiv—European Journal of Physiology 460, no. 2 (2010): 361–374. 20213496 10.1007/s00424-010-0800-x PMC2883925

[68] S. Nattel , J. Heijman , L. Zhou , et al., “Molecular Basis of Atrial Fibrillation Pathophysiology and Therapy: A Translational Perspective,” Circulation Research 127, no. 1 (2020): 51–72. 32717172 10.1161/CIRCRESAHA.120.316363 PMC7398486

[69] P. Balmaceda , T. C. Hitzeman , K. E. Shaw , et al., “Design of the First in Human Gene Therapy Trial of TLT‐101 for Chronic Heart Failure (FIGHT‐HF),” JACC: Basic to Translational Science 10, no. 3 (2025): 276–278. 40139869 10.1016/j.jacbts.2025.01.019 PMC12013832

[70] J. L. Sanchez‐Alonso , L. Fedele , J. S. Copier , et al., “Functional LTCC‐β2AR Complex Needs Caveolin‐3 and Is Disrupted in Heart Failure,” Circulation Research 133, no. 2 (2023): 120–137. 37313722 10.1161/CIRCRESAHA.123.322508 PMC10321517

[71] S. Fan and Y. Hu , “Role of m6A Methylation in the Occurrence and Development of Heart Failure,” Frontiers in Cardiovascular Medicine 9 (2022): 892113. 35811741 10.3389/fcvm.2022.892113 PMC9263194

[72] A. Charest , N. Nasta , S. Siddiqui , et al., “Nanoscale Organization of Cardiac Calcium Channels Is Dependent on Thyroid Hormone Status,” The American Journal of Physiology‐Heart and Circulatory Physiology 327, no. 5 (2024): H1309–H1326. 39365674 10.1152/ajpheart.00272.2024 PMC11559645

[73] H. Subramanian and V. O. Nikolaev , “A‐Kinase Anchoring Proteins in Cardiac Myocytes and Their Roles in Regulating Calcium Cycling,” Cells 12, no. 3 (2023): 436. 36766777 10.3390/cells12030436 PMC9913689

[74] A. Njegic , C. Wilson , and E. J. Cartwright , “Targeting Ca(2 +) Handling Proteins for the Treatment of Heart Failure and Arrhythmias,” Front Physiol 11 (2020): 1068. 33013458 10.3389/fphys.2020.01068 PMC7498719

[75] J. E. Jalil , L. Gabrielli , M. P. Ocaranza , et al., “New Mechanisms to Prevent Heart Failure With Preserved Ejection Fraction Using Glucagon‐Like Peptide‐1 Receptor Agonism (GLP‐1 RA) in Metabolic Syndrome and in Type 2 Diabetes: A Review,” The International Journal of Molecular Sciences 25, no. 8 (2024): 4407. 38673991 10.3390/ijms25084407 PMC11049921

[76] P. J. Kilfoil , S. Lotteau , R. Zhang , et al., “Distinct Features of Calcium Handling and β‐adrenergic Sensitivity in Heart Failure With Preservedversus Reduced Ejection Fraction,” The Journal of Physiology 598, no. 22 (2020): 5091–5108. 32829489 10.1113/JP280425 PMC7693093

[77] P. Pliushcheuskaya and G. Künze , “Recent Advances in Computer‐Aided Structure‐Based Drug Design on Ion Channels,” The International Journal of Molecular Sciences 24, no. 11 (2023): 9226. 37298178 10.3390/ijms24119226 PMC10253043

[78] M. J. Caterina , M. A. Schumacher , M. Tominaga , et al., “The Capsaicin Receptor: A Heat‐activated Ion Channel in the Pain Pathway,” Nature 389, no. 6653 (1997): 816–824. 9349813 10.1038/39807

[79] D. Falcón , I. Galeano‐Otero , E. Calderón‐Sánchez , et al., “TRP Channels: Current Perspectives in the Adverse Cardiac Remodeling,” Frontiers in Physiology 10 (2019): 159. 30881310 10.3389/fphys.2019.00159 PMC6406032

[80] L. Ni and C. Yuan , “The Mitochondrial‐Associated Endoplasmic Reticulum Membrane and Its Role in Diabetic Nephropathy,” Oxidative Medicine and Cellular Longevity 2021 (2021): 8054817. 34777695 10.1155/2021/8054817 PMC8589504

[81] N. Tang , W. Tian , G. Y. Ma , et al., “TRPC Channels Blockade Abolishes Endotoxemic Cardiac Dysfunction by Hampering Intracellular Inflammation and Ca(2+) Leakage,” Nature Communications 13, no. 1 (2022): 7455. 10.1038/s41467-022-35242-0 PMC9718841 36460692

[82] X. T. Hu , “HIV‐1 Tat‐Mediated Calcium Dysregulation and Neuronal Dysfunction in Vulnerable Brain Regions,” Current Drug Targets 17, no. 1 (2016): 4–14. 26028040 10.2174/1389450116666150531162212 PMC4772427

[83] Y. Du , F. Wang , P. Liu , et al., “Redox Enzymes P4HB and PDIA3 Interact With STIM1 to Fine‐Tune Its Calcium Sensitivity and Activation,” The International Journal of Molecular Sciences 25, no. 14 (2024): 7578. 39062821 10.3390/ijms25147578 PMC11276767

[84] K. C. Shin , G. Ali , H. Y. Ali Moussa , et al., “Deletion of TRPC6, an Autism Risk Gene, Induces Hyperexcitability in Cortical Neurons Derived From Human Pluripotent Stem Cells,” Molecular Neurobiology 60, no. 12 (2023): 7297–7308. 37552395 10.1007/s12035-023-03527-0 PMC10657791

[85] P. Rosenberg , H. Zhang , V. G. Bryson , et al., “SOCE in the Cardiomyocyte: The Secret Is in the Chambers,” Pflugers Archiv: European journal of physiology 473, no. 3 (2021): 417–434. 33638008 10.1007/s00424-021-02540-3 PMC7910201

[86] H. N. Rubaiy , “ORAI Calcium Channels: Regulation, Function, Pharmacology, and Therapeutic Targets,” Pharmaceuticals 16, no. 2 (2023): 162. 37259313 10.3390/ph16020162 PMC9967976

[87] J. Ren , N. N. Wu , S. Wang , et al., “Obesity Cardiomyopathy: Evidence, Mechanisms, and Therapeutic Implications,” Physiological Reviews 101, no. 4 (2021): 1745–1807. 33949876 10.1152/physrev.00030.2020 PMC8422427

[88] P. Rosenberg , D. Katz , and V. Bryson , “SOCE and STIM1 Signaling in the Heart: Timing and Location Matter,” Cell Calcium 77 (2019): 20–28. 30508734 10.1016/j.ceca.2018.11.008 PMC9520449

[89] A. Ard , C. Cruz‐Cortés , X. Gan , et al., “SAR‐Guided Development of Small‐Molecule SERCA2a Activators: Discovery of Potent Indoline, Benzofuran, and Benzodioxole Analogs for Cardiovascular Applications,” The Journal of Medicinal Chemistry 68, no. 15 (2025): 16306–16330. 40702921 10.1021/acs.jmedchem.5c01192 PMC12336636

[90] L. Zhihao , N. Jingyu , L. Lan , et al., “SERCA2a: A Key Protein in the Ca2+ Cycle of the Heart Failure,” Heart Failure Reviews 25, no. 3 (2020): 523–535. 31701344 10.1007/s10741-019-09873-3

[91] A. Zaza and M. Rocchetti , “Development of Small‐molecule SERCA2a Stimulators: A Novel Class of Ino‐lusitropic Agents,” Eur Cardiol 20 (2025): e20. 40672383 10.15420/ecr.2024.52 PMC12265601

[92] E. G. Kranias and R. J. Hajjar , “Modulation of Cardiac Contractility by the Phopholamban/SERCA2a Regulatome,” Circulation Research 110, no. 12 (2012): 1646–1660. 22679139 10.1161/CIRCRESAHA.111.259754 PMC3392125

[93] Y. Nakada , A. S. Titus , W. Mizushima , et al., “p22(phox) prevents the Oxidation of SERCA2a and Stabilizes It in the Heart,” Nature Cardiovascular Research 4, no. 9 (2025): 1187–1205. 10.1038/s44161-025-00699-x PMC12436179 40903547

[94] D. Miranda Silva , R. C. I. Wüst , G. Conceição , et al., “Disturbed Cardiac Mitochondrial and Cytosolic Calcium Handling in a Metabolic Risk‐related Rat Model of Heart Failure With Preserved Ejection Fraction,” Acta Physiologica 228, no. 3 (2020): e13378. 31520455 10.1111/apha.13378 PMC7064935

[95] B. M. Rodriguez , A. Domínguez‐Rodríguez , J. P. Benitah , et al., “Activation of Sarcolipin Expression and Altered Calcium Cycling in LMNA Cardiomyopathy,” Biochemistry and Biophysics Reports 22 (2020): 100767. 32490213 10.1016/j.bbrep.2020.100767 PMC7261707

[96] M. Periasamy , S. K. Maurya , S. K. Sahoo , et al., “Role of SERCA Pump in Muscle Thermogenesis and Metabolism,” Compr Physiol 7, no. 3 (2017): 879–890. 28640447 10.1002/cphy.c160030

[97] C. E. Molina , I. H. Abu‐Taha , Q. Wang , et al., “Profibrotic, Electrical, and Calcium‐Handling Remodeling of the Atria in Heart Failure Patients with and without Atrial Fibrillation,” Frontiers in Physiology 9 (2018): 1383. 30356673 10.3389/fphys.2018.01383 PMC6189336

[98] Z. Liu , Y. Zhang , C. Qiu , et al., “Diabetes Mellitus Exacerbates Post‐myocardial Infarction Heart Failure by Reducing Sarcolipin Promoter Methylation,” ESC Heart Failure 7, no. 4 (2020): 1935–1948. 32525286 10.1002/ehf2.12789 PMC7373908

[99] A. R. Marks , “Targeting Ryanodine Receptors to Treat human Diseases,” Journal of Clinical Investigation 133, no. 2 (2023): e162891. 36647824 10.1172/JCI162891 PMC9843046

[100] L. O. Go , M. C. Moschella , J. Watras , et al., “Differential Regulation of Two Types of Intracellular Calcium Release Channels During End‐stage Heart Failure,” The Journal of clinical investigation 95, no. 2 (1995): 888–894. 7860772 10.1172/JCI117739 PMC295578

[101] J.‐P. Benitah , R. Perrier , J.‐J. Mercadier , et al., “RyR2 and Calcium Release in Heart Failure,” Frontiers in Physiology 12 (2021): 734210. 34690808 10.3389/fphys.2021.734210 PMC8533677

[102] H. Wang , R. Jing , C. Trexler , et al., “Deletion of IP(3)R1 by Pdgfrb‐Cre in Mice Results in Intestinal Pseudo‐obstruction and Lethality,” Journal Gastroenterol 54, no. 5 (2019): 407–418. 10.1007/s00535-018-1522-7 PMC8109192 30382364

[103] P. Atakpa‐Adaji and A. Ivanova , “IP(3)R at ER‐Mitochondrial Contact Sites: Beyond the IP(3)R‐GRP75‐VDAC1 Ca(2+) Funnel,” Contact (Thousand Oaks) 6 (2023): 25152564231181020. 37426575 10.1177/25152564231181020 PMC10328019

[104] D. Lee and J. H. Hong , “Physiological Overview of the Potential Link Between the UPS and Ca(2+) Signaling,” Antioxidants (Basel) 11, no. 5 (2022): 997. 35624861 10.3390/antiox11050997 PMC9137615

[105] A. P. Arruda , B. M. Pers , G. Parlakgül , et al., “Chronic Enrichment of Hepatic Endoplasmic Reticulum–mitochondria Contact Leads to Mitochondrial Dysfunction in Obesity,” Nature Medicine 20, no. 12 (2014): 1427–1435. 10.1038/nm.3735 PMC4412031 25419710

[106] A. Al‐Sayyar , M. M. Hammad , M. R. Williams , et al., “Neurotransmitters in Type 2 Diabetes and the Control of Systemic and Central Energy Balance,” Metabolites 13, no. 3 (2023): 384. 36984824 10.3390/metabo13030384 PMC10058084

[107] P. Y. Sato , J. K. Chuprun , M. Schwartz , et al., “The Evolving Impact of G Protein‐coupled Receptor Kinases in Cardiac Health and Disease,” Physiological Reviews 95, no. 2 (2015): 377–404. 25834229 10.1152/physrev.00015.2014 PMC4551214

[108] A. Kaplan , L. El‐Samadi , R. Zahreddine , et al., “Canonical or Non‐canonical, all Aspects of G Protein‐coupled Receptor Kinase 2 in Heart Failure,” Acta Physiol (Oxf) 241, no. 3 (2025): e70010. 39960030 10.1111/apha.70010 PMC11831727

[109] M. Lieu and W. J. Koch , “GRK2 and GRK5 as Therapeutic Targets and Their Role in Maladaptive and Pathological Cardiac Hypertrophy,” Expert Opinion on Therapeutic Targets 23, no. 3 (2019): 201–214. 30701991 10.1080/14728222.2019.1575363

[110] J. J. Onorato , M. E. Gillis , Y. Liu , et al., “The β‐Adrenergic Receptor Kinase (GRK2) Is Regulated by Phospholipids,” Journal of Biological Chemistry 270, no. 36 (1995): 21346–21353. 7673171 10.1074/jbc.270.36.21346

[111] C. Gatto , M. R. Rusciano , V. Visco , et al., “GRK2 and Mitochondrial Dynamics in Cardiovascular Health and Disease,” International Journal of Molecular Sciences 26 (2025): 2299. 40076919 10.3390/ijms26052299 PMC11900936

[112] G. Kayki‐Mutlu and W. J. Koch , “Novel Roles for G Protein‐coupled Receptor Kinases in Cardiac Injury and Repair,” Biochemical Society Transactions 51, no. 2 (2023): 715–724. 37013982 10.1042/BST20221317 PMC12167929

[113] C. de Lucia , L. A. Grisanti , G. Borghetti , et al., “G Protein‐coupled Receptor Kinase 5 (GRK5) Contributes to Impaired Cardiac Function and Immune Cell Recruitment in Post‐ischemic Heart Failure,” Cardiovascular Research 118, no. 1 (2022): 169–183. 33560342 10.1093/cvr/cvab044 PMC8752360

[114] K. J. McCabe and P. Rangamani , “Computational Modeling Approaches to cAMP/PKA Signaling in Cardiomyocytes,” Journal of Molecular and Cellular Cardiology 154 (2021): 32–40. 33548239 10.1016/j.yjmcc.2021.01.008

[115] G. Liu , A. Papa , A. N. Katchman , et al., “Mechanism of Adrenergic CaV1.2 Stimulation Revealed by Proximity Proteomics,” Nature 577, no. 7792 (2020): 695–700. 31969708 10.1038/s41586-020-1947-z PMC7018383

[116] N. I. Bork , C. E. Molina , and V. O. Nikolaev , “cGMP Signalling in Cardiomyocyte Microdomains,” Biochemical Society Transactions 47, no. 5 (2019): 1327–1339. 31652306 10.1042/BST20190225

[117] M. S. Sadek , E. Cachorro , A. El‐Armouche , et al., “Therapeutic Implications for PDE2 and cGMP/cAMP Mediated Crosstalk in Cardiovascular Diseases,” International Journal of Molecular Sciences 21, no. 20 (2020): 7462. 33050419 10.3390/ijms21207462 PMC7590001

[118] M. E. Anderson , J. H. Brown , and D. M. Bers , “CaMKII in Myocardial Hypertrophy and Heart Failure,” Journal of Molecular and Cellular Cardiology 51, no. 4 (2011): 468–473. 21276796 10.1016/j.yjmcc.2011.01.012 PMC3158288

[119] O. E. Reyes Gaido , L. J. Nkashama , K. L. Schole , et al., “CaMKII as a Therapeutic Target in Cardiovascular Disease,” Annual review of pharmacology and toxicology 63 (2023): 249–272. 10.1146/annurev-pharmtox-051421-111814 PMC11019858 35973713

[120] D. M. Bers , “Ca2+‐calmodulin‐dependent Protein Kinase II Regulation of Cardiac Excitation‐transcription Coupling,” Heart Rhythm 8, no. 7 (2011): 1101–1104. 21255680 10.1016/j.hrthm.2011.01.030 PMC3129479

[121] W. Peng , Y. Zhang , M. Zheng , et al., “Cardioprotection by CaMKII‐δB Is Mediated by Phosphorylation of Heat Shock Factor 1 and Subsequent Expression of Inducible Heat Shock Protein 70,” Circulation Research 106, no. 1 (2010): 102–110. 19910575 10.1161/CIRCRESAHA.109.210914 PMC2815328

[122] P. Wang , S. Xu , J. Xu , et al., “Elevated MCU Expression by CaMKIIδB Limits Pathological Cardiac Remodeling,” Circulation 145, no. 14 (2022): 1067–1083. 35167328 10.1161/CIRCULATIONAHA.121.055841 PMC8983595

[123] R. Nussinov , C.‐J. Tsai , and C. Mattos , “‘Pathway Drug Cocktail’: Targeting Ras Signaling Based on Structural Pathways,” Trends in Molecular Medicine 19, no. 11 (2013): 695–704. 23953481 10.1016/j.molmed.2013.07.009

[124] M. Ramos‐Kuri , S. H. Meka , F. Salamanca‐Buentello , et al., “Molecules Linked to Ras Signaling as Therapeutic Targets in Cardiac Pathologies,” Biological Research 54, no. 1 (2021): 23. 34344467 10.1186/s40659-021-00342-6 PMC8330049

[125] C. A. Bill , and C. M. Vines , “Phospholipase C” Advances in Experimental Medicine and Biology 1131 (2020): 215–242. 31646512 10.1007/978-3-030-12457-1_9 PMC7790445

[126] K. Demydenko , K. R. Sipido , and H. L. Roderick , “Ca2+ release via InsP3Rs Enhances RyR Recruitment During Ca2+ Transients by Increasing Dyadic [Ca2+] in Cardiomyocytes,” Journal of Cell Science 134, no. 14 (2021): jcs258671. 34125209 10.1242/jcs.258671

[127] A. Guellich , H. Mehel , and R. Fischmeister , “Cyclic AMP Synthesis and Hydrolysis in the Normal and Failing Heart,” Pflügers Archiv—European Journal of Physiology 466, no. 6 (2014): 1163–1175. 24756197 10.1007/s00424-014-1515-1

[128] C. Hage , L. Löfgren , F. Michopoulos , et al., “Metabolomic Profile in HFpEF vs HFrEF Patients,” Journal of Cardiac Failure 26, no. 12 (2020): 1050–1059. 32750486 10.1016/j.cardfail.2020.07.010

[129] K. A. De Jong and V. O. Nikolaev , “Multifaceted Remodelling of cAMP Microdomains Driven by Different Aetiologies of Heart Failure,” The FEBS Journal 288, no. 23 (2021): 6603–6622. 33415835 10.1111/febs.15706

[130] R. D'Assante , M. Arcopinto , G. Rengo , et al., “Myocardial Expression of Somatotropic Axis, Adrenergic Signalling, and Calcium Handling Genes in Heart Failure With Preserved Ejection Fraction and Heart Failure With Reduced Ejection Fraction,” ESC Heart Failure 8, no. 2 (2021): 1681–1686. 33512777 10.1002/ehf2.13067 PMC8006736

[131] M. Gotthardt and S. E. Lehnart , “SERCA2a microdomain cAMP Changes in Heart Failure With Preserved Ejection Fraction,” Cardiovascular Research 120, no. 3 (2024): 220–222. 38333928 10.1093/cvr/cvae030

[132] R. M. Blanton , “cGMP Signaling and Modulation in Heart Failure,” Journal of Cardiovascular Pharmacology 75, no. 5 (2020): 385–398. 31464774 10.1097/FJC.0000000000000749 PMC7044023

[133] D. M. Dickey , D. R. Flora , P. M. Bryan , et al., “Differential Regulation of Membrane Guanylyl Cyclases in Congestive Heart Failure: Natriuretic Peptide Receptor (NPR)‐B, Not NPR‐A, Is the Predominant Natriuretic Peptide Receptor in the Failing Heart,” Endocrinology 148, no. 7 (2007): 3518–3522. 17412809 10.1210/en.2007-0081

[134] G. Numata and E. Takimoto , “Cyclic GMP and PKG Signaling in Heart Failure,” Frontiers in Pharmacology 13 (2022): 792798. 35479330 10.3389/fphar.2022.792798 PMC9036358

[135] J. Chen , D. Wang , F. Wang , et al., “Exendin‐4 Inhibits Structural Remodeling and Improves Ca 2+ Homeostasis in Rats With Heart Failure via the GLP‐1 Receptor Through the eNOS/cGMP/PKG Pathway,” Peptides 90 (2017): 69–77. 28242257 10.1016/j.peptides.2017.02.008

[136] J. L. Sanchez‐Alonso , A. Bhargava , T. O. Hara , et al., “Microdomain‐Specific Modulation of L‐Type Calcium Channels Leads to Triggered Ventricular Arrhythmia in Heart Failure,” Circulation Research 119, no. 8 (2016): 944–955. 27572487 10.1161/CIRCRESAHA.116.308698 PMC5045818

[137] L. van Heerebeek , N. Hamdani , I. Falcão‐Pires , et al., “Low Myocardial Protein Kinase G Activity in Heart Failure with Preserved Ejection Fraction,” Circulation 126, no. 7 (2012): 830–839. 22806632 10.1161/CIRCULATIONAHA.111.076075

[138] J. W. Wegener , H. Nawrath , W. Wolfsgruber , et al., “cGMP‐Dependent Protein Kinase I Mediates the Negative Inotropic Effect of cGMP in the Murine Myocardium,” Circulation research 90, no. 1 (2002): 18–20. 11786513 10.1161/hh0102.103222

[139] B. Fiedler , S. M. Lohmann , A. Smolenski , et al., “Inhibition of Calcineurin‐NFAT Hypertrophy Signaling by cGMP‐dependent Protein Kinase Type I in Cardiac Myocytes,” Proceedings of the National Academy of Sciences 99, no. 17 (2002): 11363–11368. 10.1073/pnas.162100799 PMC123262 12177418

[140] F. Schröder , G. Klein , B. Fiedler , et al., “Single L‐type Ca(2+) Channel Regulation by cGMP‐dependent Protein Kinase Type I in Adult Cardiomyocytes From PKG I Transgenic Mice,” Cardiovascular Research 60, no. 2 (2003): 268–277. 14613856 10.1016/s0008-6363(03)00546-7

[141] R. Rastaldo , P. Pagliaro , S. Cappello , et al., “Nitric Oxide and Cardiac Function,” Life Sciences 81, no. 10 (2007): 779–793. 17707439 10.1016/j.lfs.2007.07.019

[142] J. Y. Zhang , Investigation of the effects and underlying mechanisms of the Shunxin prescription in HFpEF rats based on the cGMP‐PKG pathway. Lanzhou University, (2019).

[143] R. M. Blanton , E. Takimoto , M. Aronovitz , et al., “Mutation of the Protein Kinase I Alpha Leucine Zipper Domain Produces Hypertension and Progressive Left Ventricular Hypertrophy: A Novel Mouse Model of Age‐Dependent Hypertensive Heart Disease,” The Journals of Gerontology: Series A 68, no. 11 (2013): 1351–1355. 10.1093/gerona/glt042 PMC3805294 23657971

[144] G. G. Schiattarella , F. Altamirano , D. Tong , et al., “Nitrosative Stress Drives Heart Failure With Preserved Ejection Fraction,” Nature 568, no. 7752 (2019): 351–356. 30971818 10.1038/s41586-019-1100-z PMC6635957

[145] K. Tanaka , R. M. Wilson , E. E. Essick , et al., “Effects of Adiponectin on Calcium‐Handling Proteins in Heart Failure with Preserved Ejection Fraction,” Circulation: Heart Failure 7, no. 6 (2014): 976–985. 25149095 10.1161/CIRCHEARTFAILURE.114.001279 PMC4241144

[146] W. Shuai , B. Kong , H. Yang , et al., “Loss of Myeloid Differentiation Protein 1 Promotes Atrial Fibrillation in Heart Failure With Preserved Ejection Fraction,” ESC Heart Failure 7, no. 2 (2020): 626–638. 31994333 10.1002/ehf2.12620 PMC7160510

[147] A. Fiordelisi , G. Iaccarino , C. Morisco , et al., “NFkappaB Is a Key Player in the Crosstalk Between Inflammation and Cardiovascular Diseases,” International Journal of Molecular Sciences 20, no. 7 (2019): 1599. 30935055 10.3390/ijms20071599 PMC6480579

[148] L. H. Lund , L. Benson , U. Dahlström , et al., “Association between Use of Renin‐Angiotensin System Antagonists and Mortality in Patients with Heart Failure and Preserved Ejection Fraction,” Jama 308, no. 20 (2012): 2108. 23188027 10.1001/jama.2012.14785

[149] Y. Xiao , L. Ni , H. Shi , et al., “SAA1 deficiency Alleviates Cardiac Remodeling by inhibitingNF‐κB /p38/JNK andTGFβ /Smad Pathways,” The FASEB Journal 37, no. 5 (2023): e22911. 37022639 10.1096/fj.202201506R

[150] M. Livitsanou , E. Vasilaki , C. Stournaras , et al., “Modulation of TGFβ/Smad Signaling by the Small GTPase RhoB,” Cellular Signalling 48 (2018): 54–63. 29705334 10.1016/j.cellsig.2018.04.007

[151] M. J. Berridge , “The Inositol Trisphosphate/Calcium Signaling Pathway in Health and Disease,” Physiological Reviews 96, no. 4 (2016): 1261–1296. 27512009 10.1152/physrev.00006.2016

[152] H. Dridi , G. Santulli , J. Gambardella , et al., “IP3 receptor Orchestrates Maladaptive Vascular Responses in Heart Failure,” Journal of Clinical Investigation 132, no. 4 (2022): e152859. 35166236 10.1172/JCI152859 PMC8843748

[153] R. Szekeres , D. Priksz , R. Kiss , et al., “Therapeutic Aspects of Prunus Cerasus Extract in a Rabbit Model of Atherosclerosis‐Associated Diastolic Dysfunction,” The International Journal of Molecular Sciences 24, no. 17 (2023): 13253. 37686067 10.3390/ijms241713253 PMC10488229

[154] J. Zheng , X. Xu , Z. Zhang , et al., “Magea13 attenuates Myocardial Injury in Acute Myocardial Infarction by Inhibiting the cAMP‐PKA Signaling Pathway,” Apoptosis 30, no. 3‐4 (2025): 1042–1057. 40056358 10.1007/s10495-025-02078-0 PMC11946976

[155] L. Lai and H. Qiu , “The Physiological and Pathological Roles of Mitochondrial Calcium Uptake in Heart,” The International Journal of Molecular Sciences 21, no. 20 (2020): 7689. 33080805 10.3390/ijms21207689 PMC7589179

[156] F. Zhang , J. J. Lin , H. N. Tian , et al., “Effect of Exercise on Improving Myocardial Mitochondrial Function in Decreasing Diabetic Cardiomyopathy,” Experimental Physiology 109, no. 2 (2024): 190–201. 37845840 10.1113/EP091309 PMC10988701

[157] C. Han , J. Yang , J. Sun , et al., “Extracellular Vesicles in Cardiovascular Disease: Biological Functions and Therapeutic Implications,” Pharmacology & Therapeutics 233 (2022): 108025. 34687770 10.1016/j.pharmthera.2021.108025 PMC9018895

[158] U. Kintscher and F. Edelmann , “The Non‐steroidal Mineralocorticoid Receptor Antagonist Finerenone and Heart Failure With Preserved Ejection Fraction,” Cardiovasc Diabetol 22, no. 1 (2023): 162. 37386461 10.1186/s12933-023-01899-0 PMC10311906

[159] M. Yu , Y. Sun , X. Shan , et al., “Therapeutic Overexpression of miR‐92a‐2‐5p Ameliorated Cardiomyocyte Oxidative Stress Injury in the Development of Diabetic Cardiomyopathy,” Cellular & Molecular Biology Letters 27, no. 1 (2022): 85. 36209049 10.1186/s11658-022-00379-9 PMC9548149

[160] M. Liao , X. Long , Y. Chen , et al., “PARP9 exacerbates Apoptosis and Neuroinflammation via the PI3K Pathway in the Thalamus and Hippocampus and Cognitive Decline After Cortical Infarction,” Journal Neuroinflammation 22, no. 1 (2025): 43. 10.1186/s12974-025-03374-x PMC11844078 39980030

[161] H. Fu , D. Li , W. Shuai , et al., “Effects of Phenylacetylglutamine on the Susceptibility of Atrial Fibrillation in Overpressure‐Induced HF Mice,” The Journal of Molecular Cell Biology 44, no. 4 (2024): 149–163. 10.1080/10985549.2024.2345363 PMC11110696 38725392

[162] T. H. Kimball and T. M. Vondriska , “Metabolism, Epigenetics, and Causal Inference in Heart Failure,” Trends in Endocrinology and Metabolism 31, no. 3 (2020): 181–191. 31866216 10.1016/j.tem.2019.11.009 PMC7035178

[163] Q. Zhang , L. Zhang , G. Lin , et al., “The Protective Role of Vagus Nerve Stimulation in Ischemia‐reperfusion Injury,” Heliyon 10, no. 10 (2024): e30952. 38770302 10.1016/j.heliyon.2024.e30952 PMC11103530

[164] C. A. Piantadosi and H. B. Suliman , “Transcriptional Control of Mitochondrial Biogenesis and Its Interface With Inflammatory Processes,” Biochimica Et Biophysica Acta 1820, no. 4 (2012): 532–541. 22265687 10.1016/j.bbagen.2012.01.003 PMC3307899

[165] N. Menghoum , M. C. Badii , M. Deltombe , et al., “Carbohydrate Antigen 125: A Useful Marker of Congestion, Fibrosis, and Prognosis in Heart Failure With Preserved Ejection Fraction,” ESC Heart Failure 11, no. 3 (2024): 1493–1505. 38339764 10.1002/ehf2.14699 PMC11098669

[166] J. M. Leser , A. Harriot , H. V. Buck , et al., “Aging, Osteo‐Sarcopenia, and Musculoskeletal Mechano‐Transduction,” Front Rehabil Sci 2 (2021): 782848. 10.3389/fresc.2021.782848 PMC9396756 36004321

[167] W. Guo , W. Guo , B. Chen , et al., “NEXN Protects Against Vascular Calcification by Promoting SERCA2 SUMOylation and Stabilization,” Nature Communications 16, no. 1 (2025): 8074. 10.1038/s41467-025-63462-7 PMC12397270 40883305

[168] W. Wang , B. Ke , C. Wang , et al., “Targeting Ion Channel Networks in Diabetic Kidney Disease: From Molecular Crosstalk to Precision Therapeutics and Clinical Innovation,” Front Med (Lausanne) 12 (2025): 1607701. 40641971 10.3389/fmed.2025.1607701 PMC12241016

[169] V. Chopra and S. Zieroth , “iCARDIO Alliance Global Implementation Guidelines on Heart Failure 2025,” Cardiovasc J Afr 36, no. 2 (2025): 35–62. 40778878 10.4081/cardio.2025.70

[170] M. Riccardi , A. M. Sammartino , M. Adamo , et al., “Cardiac Contractility Modulation: An Effective Treatment Strategy for Heart Failure Beyond Reduced Left Ventricular Ejection Fraction?,” Heart Failure Reviews 28, no. 5 (2023): 1141–1149. 37198505 10.1007/s10741-023-10315-4

[171] X. Wang , X. Zhuo , J. Gao , et al., “Neuregulin‐1β Partially Improves Cardiac Function in Volume‐Overload Heart Failure through Regulation of Abnormal Calcium Handling,” Frontiers in pharmacology 10 (2019): 616. 31281251 10.3389/fphar.2019.00616 PMC6597678

[172] N. Robbins , M. Gilbert , M. Kumar , et al., “Probenecid Improves Cardiac Function in Patients with Heart Failure with Reduced Ejection Fraction in Vivo and Cardiomyocyte Calcium Sensitivity in Vitro,” Journal of the American Heart Association 7, no. 2 (2018). 10.1161/JAHA.117.007148 PMC5850150 29331959

[173] A. A. Voors , J. F. Tamby , J. G. Cleland , et al., “Effects of danicamtiv, a Novel Cardiac Myosin Activator, in Heart Failure With Reduced Ejection Fraction: Experimental Data and Clinical Results From a Phase 2a Trial,” European Journal of Heart Failure 22, no. 9 (2020): 1649–1658. 32558989 10.1002/ejhf.1933 PMC7689751

[174] A. D. Kaplan , L. Boyman , C. W. Ward , et al., “Ryanodine Receptor Stabilization Therapy Suppresses Ca(2+)‐ based Arrhythmias in a Novel Model of Metabolic HFpEF,” Journal of Molecular and Cellular Cardiology 195 (2024): 68–72. 39053573 10.1016/j.yjmcc.2024.07.006 PMC11826478

[175] H. Zhang , Y. Gao , M. Zhang , et al., “Schaftoside Improves HFpEF Through Regulation the Autophagy‐lysosome Pathway by Allosterically Targeting CaMKII‐δ,” Redox Biology 78 (2024): 103424. 39608246 10.1016/j.redox.2024.103424 PMC11629582

[176] S. Yu , Z. Sun , X. Wang , et al., “Mettl13 protects Against Cardiac Contractile Dysfunction by Negatively Regulating C‐Cbl‐mediated Ubiquitination of SERCA2a in Ischemic Heart Failure,” Science China Life Sciences 66, no. 12 (2023): 2786–2804. 37450238 10.1007/s11427-022-2351-1

[177] M. Arici , S. C. Hsu , M. Ferrandi , et al., “Selective SERCA2a Activator as a Candidate for Chronic Heart Failure Therapy,” Journal of translational medicine 22, no. 1 (2024): 77. 38243248 10.1186/s12967-024-04874-9 PMC10797746

[178] M. Raish , A. Ahmad , Y. A. Bin Jardan , et al., “Sinapic Acid Ameliorates Cardiac Dysfunction and Cardiomyopathy by Modulating NF‐κB and Nrf2/HO‐1 Signaling Pathways in Streptozocin Induced Diabetic Rats,” Biomedicine & Pharmacotherapy 145 (2022): 112412. 34768051 10.1016/j.biopha.2021.112412

[179] J. Liu , Y. Zhao , Y. Zhu , et al., “Rhynchophylline Regulates Calcium Homeostasis by Antagonizing Ryanodine Receptor 2 Phosphorylation to Improve Diabetic Cardiomyopathy,” Frontiers in Pharmacology 13 (2022): 882198. 35517784 10.3389/fphar.2022.882198 PMC9063879

[180] P. Joshi , S. Estes , D. DeMazumder , et al., “Ryanodine Receptor 2 Inhibition Reduces Dispersion of Cardiac Repolarization, Improves Contractile Function, and Prevents Sudden Arrhythmic Death in Failing Hearts,” Elife 12 (2023). 10.7554/eLife.88638 PMC10712946 38078905

[181] W. Hu , T. Xu , P. Wu , et al., “Luteolin Improves Cardiac Dysfunction in Heart Failure Rats by Regulating Sarcoplasmic Reticulum Ca2+‐ATPase 2a,” Scientific Reports 7, no. 1 (2017): 41017. 28112209 10.1038/srep41017 PMC5253630

[182] S. Lu , Y. Liang , S. Yang , et al., “Stachydrine Hydrochloride Regulates the NOX2‐ROS‐Signaling Axis in Pressure‐Overload‐Induced Heart Failure,” International Journal of Molecular Sciences 24, no. 18 (2023): 14369. 37762672 10.3390/ijms241814369 PMC10531983

[183] R. Roy , M. Schumacher Sarah , C. Murphy Haley , et al., “Therapeutic Efficacy of a Novel Pharmacologic GRK2 Inhibitor in Multiple Animal Models of Heart Failure,” JACC: Basic to Translational Science 10, no. 2 (2025): 202–217. 40131155 10.1016/j.jacbts.2024.10.008 PMC11897459

[184] X. Peng , L. Li , R. Lin , et al., “Empagliflozin Ameliorates Ouabain‐Induced Na+ and Ca2+ Dysregulations in Ventricular Myocytes in an Na+‐Dependent Manner,” Cardiovascular Drugs and Therapy 37, no. 3 (2023): 461–469. 34982348 10.1007/s10557-021-07311-x

[185] J. Hao , L. Chang , D. Wang , et al., “Periplocin Alleviates Cardiac Remodeling in DOCA‐Salt–Induced Heart Failure Rats,” Journal of Cardiovascular Translational Research 16, no. 1 (2023): 127–140. 35616880 10.1007/s12265-022-10277-2

[186] M. Abdellatif , V. Trummer‐Herbst , F. Koser , et al., “Nicotinamide for the Treatment of Heart Failure With Preserved Ejection Fraction,” Science Translational Medicine 13, no. 580 (2021): eabd7064. 33568522 10.1126/scitranslmed.abd7064 PMC7611499

[187] M. Abudureyimu , M. Yang , X. Wang , et al., “Berberine Alleviates Myocardial Diastolic Dysfunction by Modulating Drp1‐mediated Mitochondrial Fission and Ca2+ Homeostasis in a Murine Model of HFpEF,” Frontiers of Medicine 17, no. 6 (2023): 1219–1235. 37656418 10.1007/s11684-023-0983-0

[188] J. S. Neves , A. R. Leite , G. Conceição , et al., “Effects of Triiodothyronine Treatment in an Animal Model of Heart Failure With Preserved Ejection Fraction,” Thyroid® 33, no. 8 (2023): 983–996. 37140469 10.1089/thy.2022.0717

[189] L. H. Lund , C. Hage , G. Pironti , et al., “Acyl Ghrelin Improves Cardiac Function in Heart Failure and Increases Fractional Shortening in Cardiomyocytes Without Calcium Mobilization,” European Heart Journal 44, no. 22 (2023): 2009–2025. 36916707 10.1093/eurheartj/ehad100 PMC10256198

[190] A. A. Voors , S. J. Shah , J. J. Bax , et al., “Rationale and Design of the Phase 2b Clinical Trials to Study the Effects of the Partial Adenosine A1‐receptor Agonist Neladenoson Bialanate in Patients With Chronic Heart Failure With Reduced (PANTHEON) and Preserved (PANACHE) Ejection Fraction,” European Journal of Heart Failure 20, no. 11 (2018): 1601–1610. 30225882 10.1002/ejhf.1295

[191] J. S. Hulot , J. E. Salem , A. Redheuil , et al., “Effect of Intracoronary Administration of AAV1/SERCA2a on Ventricular Remodelling in Patients With Advanced Systolic Heart Failure: Results From the AGENT‐HF Randomized Phase 2 Trial,” European Journal of Heart Failure 19, no. 11 (2017): 1534–1541. 28393439 10.1002/ejhf.826

[192] H. S. Z. Bahrami , R. B. Hasselbalch , H. Søholm , et al., “First‐In‐Man Trial of β3‐Adrenoceptor Agonist Treatment in Chronic Heart Failure: Impact on Diastolic Function,” Journal Cardiovasc Pharmacol 83, no. 5 (2024): 466–473. 10.1097/FJC.0000000000001545 38452283

[193] J. Rubinstein , J. G. Woo , A. M. Garcia , et al., “Probenecid Improves Cardiac Function in Subjects With a Fontan Circulation and Augments Cardiomyocyte Calcium Homeostasis,” Pediatric Cardiology 41, no. 8 (2020): 1675–1688. 32770262 10.1007/s00246-020-02427-7 PMC7704717

[194] T. Matsumura , T. Fukudome , Y. Motoyoshi , et al., “Efficacy of tranilast in Preventing Exacerbating Cardiac Function and Death From Heart Failure in Muscular Dystrophy Patients With Advanced‐stage Heart Failure: A Single‐arm, Open‐label, Multicenter Study,” The Orphanet Journal of Rare Diseases 20, no. 1 (2025): 13. 39789553 10.1186/s13023-025-03538-1 PMC11720297

[195] X. Wang , J. Ju , Z. Chen , et al., “Associations Between Calcium Channel Blocker Therapy and Mortality in Heart Failure With Preserved Ejection Fraction,” European Journal of Preventive Cardiology 29, no. 9 (2022): 1343–1351. 35015840 10.1093/eurjpc/zwac004

[196] N. Kaneko , C. M. Loughrey , G. Smith , et al., “A Novel Ryanodine Receptor 2 Inhibitor, M201‐A, Enhances Natriuresis, Renal Function and Lusi‐inotropic Actions: Preclinical and Phase I Study,” British Journal of Pharmacology 181, no. 18 (2024): 3401–3419. 38773354 10.1111/bph.16379

[197] P. Fikar , L. Alvarez , L. Berne , et al., “Enhancing Reproducibility in Single Cell Research With Biocytometry: An Inter‐laboratory Study,” PLoS ONE 19, no. 12 (2024): e0314992. 39652549 10.1371/journal.pone.0314992 PMC11627387

[198] M. M. Cortese‐Krott , A. Koning , G. G. C. Kuhnle , et al., “The Reactive Species Interactome: Evolutionary Emergence, Biological Significance, and Opportunities for Redox Metabolomics and Personalized Medicine,” Antioxid Redox Signaling 27, no. 10 (2017): 684–712. 10.1089/ars.2017.7083 PMC5576088 28398072

[199] J. Huang , Q. Feng , L. Wang , et al., “Human Pluripotent Stem Cell‐Derived Cardiac Cells: Application in Disease Modeling, Cell Therapy, and Drug Discovery,” Frontiers in Cell and Developmental Biology 9 (2021): 655161. 33869218 10.3389/fcell.2021.655161 PMC8049435

[200] J. Gallagher , S. James , C. Keane , et al., “Heart Failure Virtual Consultation: Bridging the Gap of Heart Failure Care in the Community—A Mixed‐methods Evaluation,” ESC Heart Failure 4, no. 3 (2017): 252–258. 28772044 10.1002/ehf2.12163 PMC5542774

[201] K. K. Patel , C. Venkatesan , H. Abdelhalim , et al., “Genomic Approaches to Identify and Investigate Genes Associated With Atrial Fibrillation and Heart Failure Susceptibility,” Human Genomics 17, no. 1 (2023): 47. 37270590 10.1186/s40246-023-00498-0 PMC10239148

[202] H. T. Ong , E. Karatas , T. Poquillon , et al., “Digitalized Organoids: Integrated Pipeline for High‐speed 3D Analysis of Organoid Structures Using Multilevel Segmentation and Cellular Topology,” Nature Methods 22, no. 6 (2025): 1343–1354. 40369245 10.1038/s41592-025-02685-4 PMC12165853

[203] N. M. Mordwinkin , P. W. Burridge , and J. C. Wu , “A Review of human Pluripotent Stem Cell‐derived Cardiomyocytes for High‐throughput Drug Discovery, Cardiotoxicity Screening, and Publication Standards,” Journal of Cardiovascular Translational Research 6, no. 1 (2013): 22–30. 23229562 10.1007/s12265-012-9423-2 PMC3556463

[204] A. Hammerich , “How Are Countries Dealing With Their Current Cardio‐vascular Disease Burden? A Snapshot From the WHO Eastern Mediterranean Region (EMR),” Global Cardiology Science & Practice 2018, no. 1 (2018): 1. 29644228 10.21542/gcsp.2018.1 PMC5857060

[205] C. Ahn , B.‐S. An , and E.‐B. Jeung , “Streptozotocin Induces Endoplasmic Reticulum Stress and Apoptosis via Disruption of Calcium Homeostasis in Mouse Pancreas,” Molecular and Cellular Endocrinology 412 (2015): 302–308. 26003140 10.1016/j.mce.2015.05.017

[206] D. Zhang , F. Wang , P. Li , et al., “Mitochondrial Ca2+ Homeostasis: Emerging Roles and Clinical Significance in Cardiac Remodeling,” International Journal of Molecular Sciences 23, no. 6 (2022): 3025. 35328444 10.3390/ijms23063025 PMC8954803

[207] S. Gao , X. Yao , J. Chen , et al., “Structural Basis for human Cav1.2 Inhibition by Multiple Drugs and the Neurotoxin Calciseptine,” Cell 186, no. 24 (2023): 5363–5374.e5316. 37972591 10.1016/j.cell.2023.10.007

[208] H. Morikawa , C. C. Young , and J. A. Smits , “Usage of L‐type Calcium Channel Blockers to Suppress Drug Reward and Memory Driving Addiction: Past, Present, and Future,” Neuropharmacology 221 (2022): 109290. 36241085 10.1016/j.neuropharm.2022.109290 PMC10476140

[209] C. Kho , “Targeting Calcium Regulators as Therapy for Heart Failure: Focus on the Sarcoplasmic Reticulum Ca‐ATPase Pump,” Frontiers in Cardiovascular Medicine 10 (2023): 1185261. 37534277 10.3389/fcvm.2023.1185261 PMC10392702

[210] S. Tang , X. Deng , J. Jiang , et al., “Design of Calcium‐Binding Proteins to Sense Calcium,” Molecules (Basel, Switzerland) 25, no. 9 (2020): 2148. 32375353 10.3390/molecules25092148 PMC7248937

[211] J. Jin , S. Qin , Q. Fu , et al., “Identification of Biomarkers and Immune Microenvironment Associated With Heart Failure Through Bioinformatics and Machine Learning,” Frontiers in Molecular Biosciences 12 (2025): 1580880. 40406620 10.3389/fmolb.2025.1580880 PMC12095026

[212] X. Zhang , W. Yu , Y. Li , et al., “Drug Development Advances in human Genetics‐based Targets,” MedComm 5, no. 2 (2024): e481. 38344397 10.1002/mco2.481 PMC10857782

[213] E. Toni , H. Ayatollahi , R. Abbaszadeh , et al., “Machine Learning Techniques for Predicting Drug‐Related Side Effects: A Scoping Review,” Pharmaceuticals (Basel) 17, no. 6 (2024): 795. 38931462 10.3390/ph17060795 PMC11206653

[214] Y. Qi , J. Yin , W. Xia , et al., “Exploring the Role of Mitochondrial Antiviral Signaling Protein in Cardiac Diseases,” Frontiers in Immunology 16 (2025): 1540774. 40040697 10.3389/fimmu.2025.1540774 PMC11876050

